# Effects of 6-month customized home-based exercise on motor development, bone strength, and parental stress in children with simple congenital heart disease: a single-blinded randomized clinical trial

**DOI:** 10.1186/s12916-023-03242-6

**Published:** 2024-02-06

**Authors:** Qing Du, Xin Li, Zhaoxi Wang, Sun Chen, Xi Zhang, Juping Liang, Haibin Guo, Nan Chen, Hong Yu, Xiaoqing Zhu, Xuan Zhou, Kun Sun

**Affiliations:** 1 Department of Rehabilitation Medicine, Xinhua Hospital, School of Medicine, Shanghai Jiao Tong University, Shanghai, China; 2 School of Exercise and Health, Shanghai University of Sport, Shanghai, China; 3 Beth Israel Deaconess Medical Center, Boston, MA USA; 4 Harvard Medical School, Boston, MA USA; 5 Department of Pediatric Cardiology, Xinhua Hospital, School of Medicine, Shanghai Jiao Tong University, Shanghai, China; 6 Clinical Research Unit, Xinhua Hospital Affiliated to Shanghai Jiaotong University School of Medicine, Shanghai, China

**Keywords:** Congenital heart disease, Customized training program, Children, Home-based exercise, Cardiac catheterization

## Abstract

**Background:**

New “noncardiac” problems in children with congenital heart disease (CHD), such as developmental delay or long-term neurodevelopmental impairments, have attracted considerable attention in recent years. It is hypothesized that exercise might attenuate CHD-associated neurodevelopmental impairments; however, this has not been thoroughly investigated. The objective of this prospective, single-blinded, randomized controlled experiment was to evaluate the impact of customized home-based exercise for children with CHD.

**Methods:**

Children aged 0–5 years with echocardiography-confirmed simple CHD subtypes who were scheduled to undergo cardiac catheterization were screened for enrolment. Among 420 screened CHD children, 192 were enrolled and randomly assigned at a 1:1 ratio to receive a 6-month intervention (30 min daily customized home-based exercise program with supervision for no less than 5 days per week, combined with home-based exercise education) or control treatment (home-based education). The primary outcome was motor development (gross motor quotient (GMQ), fine motor quotient (FMQ), and total motor quotient (TMQ)). The secondary outcomes were cardiac function and structure, bone quality, physical development, parental anxiety, caregiver burden, and quality of life. Children and their families were assessed before and 1, 3, and 6 months after catheterization; 183 (95.3%) children were included in the primary analysis.

**Results:**

After 6-month treatment, the intervention group significantly increased their motor quotient, which was consistently higher than that of the control group (GMQ *p* < 0.0001, FMQ *p* = 0.02, TMQ *p* < 0.001). The physical developments in height, weight, and circumferences of the upper-arm, chest, and head were also significantly improved by exercise (all *p* < 0.017). No significant improvements in the bone strength or the cardiac structure and function were found among patients in the intervention group (all *p* > 0.017). For parents, higher quality of life level (total score *p* = 0.016) was observed in the intervention group; while effects of exercise on the anxiety (rude score *p* = 0.159, standard score *p* = 0.159) or the Zarit caregiver burden scale score (*p* = 0.404) were non-significant. No adverse events occurred during the study period.

**Conclusions:**

Customized home-based exercise improved motor development in children with CHD. While the long-term effects of parent training in home-based exercise are unknown, the study results suggest positive outcomes.

**Trial registration:**

A home-based exercise program in congenital heart disease children with cardiac catheterization: a randomized controlled trial. (http://www.chictr.org.cn/, ChiCTR-IOR-16007762, January 14, 2016).

**Supplementary Information:**

The online version contains supplementary material available at 10.1186/s12916-023-03242-6.

## Background

Congenital heart disease (CHD) is one of the most common structural abnormalities of the cardiovascular system, occurring in 9.41 of every 1000 live births [1]. Advances in pediatric health care over recent decades, such as approaches in pediatric cardiac catheterization, have enabled the majority of children with CHD (more than 97%) to enter adulthood successfully [2]. In recent years, new “noncardiac” problems in children with CHD [2, 3], such as developmental delay or long-term neurodevelopmental impairments characterized by delayed motor development, cognitive impairments, and stunted growth, have been recognized [4, 5].

Studies have shown that the period from 0 to 5 years of age is a critical period for heart development, with rapid growth in size and weight [6]. Strong evidence suggests that infants and toddlers under 5 years old should carry out unstructured physical activity every day in line with the characteristics of motor development [7, 8], and compared with other periods, reasonable physical activity at this stage can promote the development of heart and lung function, promote bone health and brain development, and provide lifelong benefits [9]. However, many studies have pointed out that children with CHD have lower levels of physical activity than healthy children of the same age, and this gap is more significant in infancy [9]. Impaired sensory and motor development may further negatively affect a child’s psychosocial development, resulting in further isolation from peers [10]. The lack of physical activity in children with CHD may be related to excessive parental protection, which has been reported as a contributing factor to stunted growth in children with CHD [10, 11]. Although there is strong evidence that early exercise interventions can help children recover more quickly, reduce the risks of developmental delay, and prevent stunting [12, 13], patient-reported limitations in sports participation are common, resulting in markedly reduced physical activity and a sedentary lifestyle with an increased risk of related complications [14–16]. Moreover, the lack of physical activity reduces social interactions, and delayed development can cause school-aged children with CHD children to adapt poorly to school life, resulting in poor academic performance. These problems may persist into young adulthood, leading to a low quality of life for these children and their families [17].

Although exercise training is safe and effective and abundant scientific evidence has demonstrated that physically active people of all ages and ethnicities have higher levels of cardiorespiratory fitness [18–20], the majority of children with CHD only receive very limited access to rehabilitation facilities, indicating that these services need to be arranged more effectively [21]. Moreover, the COVID-19 pandemic has led to social distancing and relatively few direct interactions between healthcare professionals and patients; therefore, patients who need intensive interactions with healthcare professionals have suffered to a greater extent [22]. Home-based cardiac rehabilitation has been widely used in adults and adolescents with CHD, and numerous studies have confirmed that home-based cardiac rehabilitation is safe and feasible and has high patient compliancee [23–26]. Focusing rehabilitation services on home-based exercise, training and expert supervision of parents may be an effective way to ensure positive outcomes for children with CHD, especially during the pandemic. A preliminary study showed that a customized home-based exercise program was beneficial for very young children [27]; however, high-quality randomized controlled trials are urgently needed. This randomized controlled trial was conducted to evaluate the impact of a customized home-based exercise program on (1) the motor abilities of children with CHD after cardiac catheterization; (2) the bone quality, cardiac function and structure, and physical development of children with CHD after cardiac catheterization; (3) parental anxiety and caregiver burden; and (4) parental quality of life.

## Methods

### Participants

This study was a prospective, single-blinded, randomized controlled trial conducted at Xinhua Hospital affiliated to Shanghai Jiaotong University School of Medicine from January 2016 to December 2019. It was approved by the Ethics Committee, which is recognized by the Strategic Initiative for Developing Capacity in Ethical Review in collaboration with the Forum for Ethical Review Committees in Asia and the Western Pacific Region (Chinese Clinical Trial Registry: ChiCTR-IOR-16007762, January 14, 2016). The full protocol, including detailed descriptions of the intervention and statistical analysis plan, has been published previously (in the supplementary material) [28]. Patients and the public were not involved in the design, conduct, reporting, or dissemination plans of this research.

Study participants were recruited from the outpatient clinic, and the disease severity was classified with the categories “simple CHD,” “CHD of moderate complexity,” and “CHD of great complexity” using the system adopted by the Bethesda conference [29]. The inclusion criteria were children who (1) had echocardiography findings confirming a simple CHD subtype, including patent ductus arteriosus (PDA), pulmonary stenosis (PS), ventricular septal defect (VSD), and atrial septal defect (ASD); (2) children who were 0–5 years old; and (3) children who were scheduled to undergo cardiac catheterization. The exclusion criteria were (1) children with CHD of moderate and high complexity; (2) children with CHD combined with arrhythmia; (3) children with CHD combined with genetic disorders or other congenital musculoskeletal deformities; (4) children with CHD combined with liver or kidney diseases; (5) children with CHD combined with heart failure with a modified Ross score ≥ 3; (6) children with a history of heart surgery; (7) children with a history of surgery on other organs; (8) children who received previous rehabilitation treatment; and (9) children with illnesses that may preclude their participation as identified by the study physician [30]. Each participant’s parents signed a written informed consent form before participating in the trial.

### Randomization and blinding

Randomization was computer-generated with allocation concealment by opaque sequentially numbered sealed envelopes. Eligible participants were randomized to receive either a home-based exercise program (an add-on home-based exercise program with supervision in addition to home-based exercise education) or home-based exercise education alone in a 1:1 allocation ratio. Outcome assessors were blinded to group allocation.

### Procedures

In the intervention group, each patient and their parents participated in a home-based exercise program consisting of home-based exercise with supervision and home-based exercise education [28]. The degree of developmental delay and the achievement of age-appropriate skills were identified using the Peabody Developmental Motor Scales, 2nd edition (PDMS-2) before catheterization. Taking the degree of developmental delay, developmental age according to age-appropriate skill achievement, and CHD severity into consideration for each child, an individualized home-based exercise protocol was jointly designed by a multidisciplinary team, including a pediatric cardiologist, rehabilitation physician, and physical therapists specializing in pediatric rehabilitation, with input from the parents. The individualized home-based exercise protocol was then adjusted as the child’s skills developed at 1 and 3 months postcatheterization.

Parents received one-on-one personalized guidance until they understood and were able to demonstrate the capability of completing at least one whole session of the exercise program. The parents of participants at all included stages of growth were advised to incorporate these activities and preferred behaviors into their daily schedules (Additional file 1: Table S1). At least one of the children’s parents was asked to complete the entire exercise program at home 30 min daily for no less than 5 days per week over a 6-month period, with a weekly reminder from the intervention team, and compliance was recorded based on parent feedback through a Micro Message Public Platform or phone call one or two times per week. The intervention team also maintained phone contact with the parents to provide exercise guidance.

Both the intervention and control groups received the same home-based exercise education program, which consisted of an explanation of the precatheterization assessment results and educational materials. The educational materials were released through a WeChat subscription account twice monthly, which shared various forms of CHD knowledge, including approaches and matters needing attention regarding exercise and general care. Daily outdoor activities were also recommended.

### Outcomes

Each child was assessed before catheterization and at 1, 3, and 6 months after surgical catheterization by the evaluation team that was blinded to the group assignments. The age range between 0 and 5 years old is the most rapid developmental period, and surgical procedures during this time can delay regular growth [31]. The primary outcome was motor development, including the gross motor quotient (GMQ), fine motor quotient (FMQ), and total motor quotient (TMQ). Secondary outcomes included bone strength examined by quantitative ultrasound at the tibia, cardiac structural indexes determined by echocardiography and the modified Ross score, and physical development indicators. Children’s physical development indicators were also evaluated as additional secondary outcomes, including height, weight, body mass index (BMI), head circumference, chest circumference, and upper arm circumference. We also measured parental anxiety, caregiver burden, and parental quality of life at each assessment using the Self-Rating Anxiety Scale (SAS) [32], a modified version of the Zarit Caregiver Burden Scale (ZCBS) [33, 34], and the 36-item Short-Form Health Survey (SF-36) [35]. Adverse effects occurring during the trial and any event that could be related to the exercise performed, such as abnormal heart failure or bone fracture, were considered adverse events (AEs). AEs were monitored and reported directly to the researchers by parents using phone or WeChat.

### Statistical analysis

Normality tests were initially performed on the data. Nonnormally distributed data are reported as medians with interquartile ranges (IQRs) and were compared between the intervention group and the control group using an independent sample nonparametric test (Mann–Whitney *U* test). Data were also compared between the baseline assessment and each follow-up assessment within each group using a matched sample nonparametric test (Wilcoxon rank sum test). Categorical data are reported as numbers with percentages and were analyzed using the chi-squared test. Mixed-model analysis was used to compare the primary and secondary outcomes between the intervention and control groups among the four assessments. *Z* scores for speed of sound (SOS) are used to represent the difference between each patient’s value and the age-specific mean value divided by the standard deviation of the reference group, which was based on data for age-matched SOS values from the manufacturer’s data bank (not differentiated by race) [36]. We used intention-to-treat (ITT) analysis according to group allocation as the main analytical method in this study and included all patients who were randomly assigned, regardless of whether they received treatment. Statistical analysis was performed using the software package SPSS 25.0, and GPower 3.1.9.2 was used for sample size estimation. *P* values of < 0.017 were considered statistically significant, while the Bonferroni method was carried out to correct multiple comparison.

## Results

**Fig. 1:**
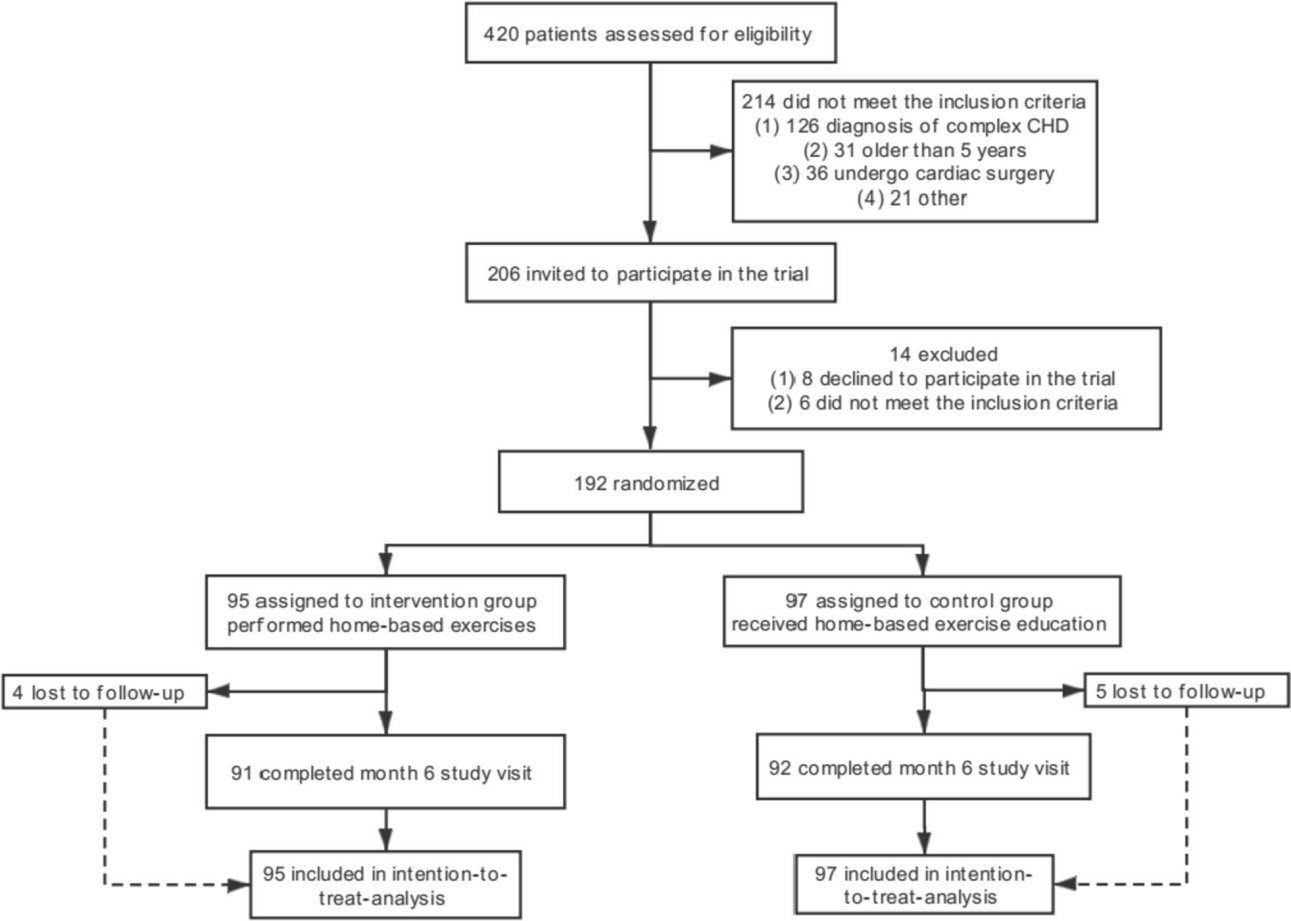
CONSORT diagram. Prerecruitment assessments were performed in 420 children, resulting in 228 children being excluded before recruitment and 192 children being included in the study. At the end of the study, 91 children in the intervention group and 92 patients in the control group completed the 6-month follow-up. Intention-to-treat (ITT) analysis was performed including the 95 originally recruited children in the intervention group and 97 patients in the control group. No adverse events were reported during this study

Prerecruitment assessments were performed for 420 children with CHD, resulting in 228 children being excluded before recruitment. Of these children, 214 children did not meet the inclusion criteria, the parents of 8 children declined to participate in the trial, and 6 children were excluded for not meeting the inclusion criteria in the final preenrolment evaluation. We included a total of 192 children with CHD, including 64 with PDA, 26 with PS, 64 with VSD, and 38 with ASD (Fig. 1). There were no significant differences in baseline characteristics between the intervention (*n* = 95) and control (*n* = 97) groups (Table 1). Although most patient families (~ 70%) were from other regions of China and had to arrange family travel to Shanghai for the 3 follow-up assessments, 183 children (retention rate: 95%) completed the 6-month rehabilitation study, including 92 in the intervention group and 91 in the control group. The reasons for drop-out were attributed to parent work relocations (*n* = 2) and personal matters (*n* = 7). No serious AEs were reported during the study.

**Table 1:** Preoperative assessment of the enrolled children with CHD

	Intervention group (*n* = 95)	Control group (*n* = 97)
Age, median month (IQR)	33.0 (16.0–47.0)	33.0 (16.5–44.0)
Shanghai residency	30 (31.6)	27 (27.8)
Gender, male (%)	36 (37.9)	49 (50.5)
CHD subtypes, *n* (%)		
PDA	31 (32.6)	32 (33.0)
PS	12 (12.6)	14 (14.4)
VSD	33 (34.7)	32 (33.0)
ASD	19 (20.0)	19 (19.6)
Motor developmental quotient, median (IQR)		
GMQ	91.0 (85.0–94.0)	91.0 (85.0–97.0)
FMQ	94.0 (91.0–100.0)	97.0 (91.0–100.0)
TMQ	92.0 (86.0–96.0)	92.0 (89.0–97.5)
Modified Ross score, *n* (%)		
0	37 (38.9)	39 (40.2)
1	45 (47.4)	44 (45.4)
2	13 (13.7)	14 (14.4)
Cardiac structural indexes, median (IQR)		
LVDd, mm	33.3 (29.2–36.3)	32.6 (29.4–36.1)
Z score of LVDd	0.16 (− 0.63–1.42)	0.14 (− 0.50–1.21)
LVDs, mm	21.2 (18.7–23.1)	20.8 (18.6–23.3)
Z score of LVDs	0.46 (− 0.31–1.36)	0.38 (− 0.27–1.34)
LVPWd, mm	4.6 (4.0–5.1)	4.4 (3.7–5.0)
Z score of LVPWd	0.36 (− 0.20–0.99)	0.16 (− 0.46–0.77)
LVPWs, mm	7.6 (7.0–9.0)	7.3 (6.4–8.5)
Z score of LVPWs	− 0.27 (− 1.11–0.54)	− 0.58 (− 1.45–0.20)
LVEF, %	69.0 (64.5–71.0)	66.0 (63.5–70.0)
MPA, %	1.2 (1.0–1.4)	1.2 (1.0–1.4)
MV, m/s	1.0 (1.0–1.2)	1.0 (1.0–1.2)
TV, m/s	0.8 (0.8–1.0)	0.8 (0.8–1.0)
Z score of LVPWs	− 0.27 (− 1.11–0.54)	− 0.58 (− 1.45–0.20)
Bone quality, median (IQR)		
SOS, m/s	3445.0 (3233.0–3543.5)	3382.0 (3261.0–3516.0)
Bone **s**trength percentile	55.0 (31.0–78.0)	44.0 (20.0–76.5)
*Z* score of SOS	0.1 (− 0.5–0.8)	− 0.2 (− 0.85–0.75)
SAS score, median (IQR)		
Rude score	30.0 (26.5–37.0)	33.0 (27.0–39.0)
Standard score	37.5 (33.1–46.3)	41.3 (33.8–48.8)
ZCBS score, median (IQR)	22.0 (14.5–30.0)	22.5 (14.3–30.0)
SF-36 score, median (IQR)		
PCS	339.0 (304.0–369.0)	348.0 (288.0–370.0)
MCS	308.0 (242.5–340.0)	277.0 (237.5–327.0)
Total score	640.0 (552.5–706.5)	629.0 (532.5–686.0)
Physical development indicators, median (IQR)		
Weight, kg	14.0 (10.0–17.0)	13.5 (9.6–16.7)
Height, cm	93.0 (79.0–104.0)	92.5 (78.0–100.0)
BMI, kg/m^2^	16.3 (15.4–17.7)	16.1 (15.0–17.3)
Upper-arm circumference, cm	16.0 (15.0–17.0)	16.0 (14.9–17.0)
Head circumference, cm	48.3 (45.0–50.0)	47.7 (46.0–49.5)
Chest circumference, cm	50.5 (47.0–53.5)	50.0 (47.0–54.0)

*Abbreviations*: *Ao* Aort, *ASD* Atrial septal defect, *BMI* Body mass index, *CHD* Congenital heart disease, *FMQ* Fine motor quotient, *GMQ* Gross motor quotient, *LVDd* Left ventricular end diastolic dimension, *LVDs* Left ventricular end-systolic dimension, *LVEF* Left ventricular ejection fraction, *LVPWd* Left ventricular posterior wall depth, *LVPWs* Left ventricular posterior wall thickness at end-systole, *MCS* Mental component summary, *MPA* Main pulmonary artery diameter, *MV* Mitral blood flow velocity, *PCS* Physical component summary, *PDA* Patent ductus arteriosus, *PS* Pulmonary stenosis, SAS Self-rating anxiety scale, *SF-36* 36-item short-form health survey, *SOS* Speed of sound, *TMQ* Total motor quotient, *TV* Tricuspid blood flow velocity, *VSD* Ventricular septal defect, *ZCBS* Zarit caregiver burden scale

**Fig. 2:**
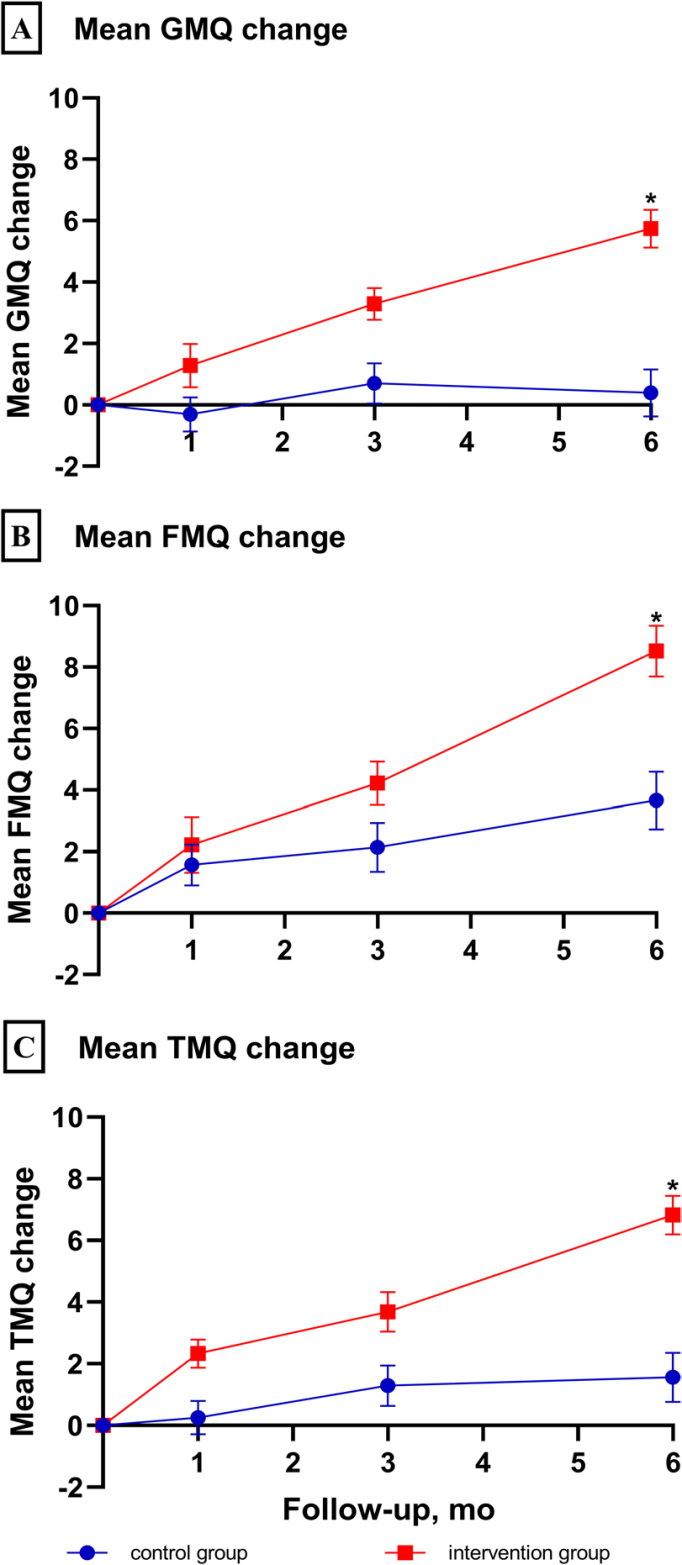
Postcatheterization changes in motor development quotients. The *X*-axis shows the time points (months) of the follow-up, with “0” representing the preoperative assessment. The *Y*-axis shows the mean changes in the values of the developmental quotients, as calculated by subtracting each of the preoperative values from the postoperative assessment values. GMQ indicates gross motor quotient. FMQ indicates fine motor quotient. TMQ indicates total motor quotient. * indicates that the GMQ, FMQ, and TMQ scores of the intervention group were significantly greater than those of the control group at the 6-month assessment (*p* < 0.017)

The trends of motor quotients after 6 months, within and between the treatment groups, were examined (Fig. 2). The intervention group demonstrated a trend of increasing motor quotients over the course of the study, whereas the control group demonstrated relatively small increases in both the FMQ and TMQ. This difference in motor quotient trends between the groups resulted in the mean postcatheterization change in the GMQ at the 6-month assessment being nearly 15-fold higher in the intervention group (5.74; 95% confidence interval (CI) 4.52–6.96) than in the control group (0.39; 95% CI − 1.14–1.92). The GMQ of the control group decreased 1 month postcatheterization and returned to baseline after 6 months (Fig. 2).

Next, we compared the pre- and postcatheterization assessments for each group. For the intervention group, all motor quotients had significantly increased at 1 month postcatheterization (Wilcoxon rank sum test for paired samples, *p* < 0.001 in all motor quotients, Table 2). In contrast, the control group only had a significant increase in the FMQ in the first month postcatheterization and no significant changes at any follow-up measurements for the GMQ and TMQ. Furthermore, the GMQ, FMQ, and TMQ of the intervention group were significantly higher than those of the control group (mixed modeling: _*fixed effect*_ = 2.52, 95% CI 1.35–3.70; _*fixed effect*_ = 2.31, 95% CI 0.83–3.79; and _*fixed effect*_ = 2.80, 95% CI 1.74–3.86, respectively, Table 3).

**Table 2:** The Wilcoxon rank sum test of primary outcomes (motor development quotients) against preoperative assessment

	Intervention group(*n* = 95)		Control group(*n* = 97)	
	Median (IQR)	*p* value	Median (IQR)	*p* value
Gross motor quotient (GMQ)				
Baseline	91.0 (85.0–94.0)	-	91.0 (85.0–97.0)	-
1 month	91.0 (85.0–94.0)	< 0.001*	91.0 (85.0–96.0)	0.461
3 months	94.0 (89.0–98.0)	< 0.001*	91.0 (87.0–96.0)	0.455
6 months	96.0 (91.0–100.0)	< 0.001*	91.0 (87.0–97.0)	0.780
Fine motor quotient (FMQ)				
Baseline	94.0 (91.0–100.0)	-	97.0 (91.0–100.0)	-
1 month	97.0 (94.0–103.0)	< 0.001*	97.0 (91.0–103.0)	0.013*
3 months	100.0 (947.0–106.0)	< 0.001*	97.0 (94.0–103.0)	0.016*
6 months	106.0 (100.0–109.0)	< 0.001*	100.0 (94.0–106.0)	0.001*
Total motor quotient (TMQ)				
Baseline	92.0 (86.0–96.0)	-	92.0 (89.0–97.5)	-
1 month	93.0 (90.0–98.0)	< 0.001*	92.0 (89.0–97.0)	0.640
3 months	96.0 (92.0–100.0)	< 0.001*	93.0 (89.0–97.0)	0.145
6 months	100.0 (94.0–103.0)	< 0.001*	94.0 (90.0–99.0)	0.122

In this table, we compared postoperative assessments against the preoperative assessment separately in the intervention group and in the control group

*IQR* the interquartile range

^*^means significant difference (*p* < 0.017)

**Table 3:** Mixed-model analysis of primary and secondary outcomes between the intervention and the control group (*n* = 192)

	*F* value	95% CI	*p* value
Primary outcomes			
Gross motor quotient (GMQ)	2.52	1.35–3.70	< 0.0001*
Fine motor quotient (FMQ)	2.31	0.83–3.79	0.002*
Total motor quotient (TMQ)	2.80	1.74–3.86	< 0.0001*
Secondary outcomes			
Ross score	0.07	− 0.04–0.17	0.217
SOS (m/s)	− 16.84	− 41.60–7.93	0.182
Bone strength percentile	− 0.83	− 5.60–3.93	0.731
*Z* score of SOS	0.10	− 0.15–0.36	0.431
Weight (kg)	0.21	− 0.09–0.52	0.175
Height (cm)	0.23	− 0.58–1.05	0.571
BMI (kg/m^2^)	0.15	− 0.18–0.47	0.371
Upper-arm circumference (cm)	0.35	0.05–0.65	0.021
Head circumference (cm)	0.15	− 0.17–0.48	0.364
Chest circumference (cm)	0.48	− 0.14–1.12	0.126

In this table, we compared trends of primary and secondary outcomes between the intervention and the control group using mixed effects modeling. *F* values of fixed effects were reported

*BMI* Body mass index, *SOS* Speed of sound

^*^ means significant difference (*p* < 0.017)

The secondary outcomes of bone strength, including the bone mineral density (BMD) percentiles and Z score of SOS, were not significantly different at any stage of the study compared with the precatheterization assessment within either the intervention or control group (Wilcoxon rank sum test for paired samples, *p* > 0.017, Table 4). In contrast, there was a significant increase in the SOS in the control group at the 6-month postcatheterization assessment (*p* = 0.001; Table 4). There was no significant difference in the SOS between the two groups pre- and posttreatment (mixed model: _*fixed effect*_ =  − 16.84, 95% CI − 41.60 7.93; Additional file 1: Table S3).

**Table 4:** The Wilcoxon rank sum test of secondary outcomes (bone strength and physical development indicators) against preoperative assessment

	Intervention group (*n* = 95)		Control group (*n* = 97)	
	Median (IQR)	*p* value	Median (IQR)	*p* value
SOS (m/s)				
Baseline	3445.0 (3233.0–3543.5)	-	3373.3 (3240.0–3516.0)	-
1 month	3407.0 (3269.0–3523.5)	0.969	3386.0 (3247.0–3529.5)	0.601
3 months	3438.0 (3265.0–3528.0)	0.614	3410.0 (3240.0–3544.0)	0.083
6 months	3407.0 (3264.5–3545.0)	0.239	3425.0 (3287.0–3545.0)	0.001^*^
Bone mineral density (BMD) percentiles				
Baseline	55.0 (31.0–78.0)	-	41.0 (19.5–75.0)	-
1 month	58.0 (24.0–85.0)	0.957	47.0 (18.0–81.5)	0.868
3 months	54.0 (22.5–78.5)	0.350	47.0 (17.5–83.0)	0.615
6 months	50.0 (27.5–77.0)	0.343	49.0 (26.0–83.0)	0.261
*Z* score of SOS				
Baseline	0.1 (− 0.5–0.8)	-	− 0.2 (− 0.9–0.7)	-
1 month	0.2 (− 0.7–1.0)	0.809	− 0.2 (− 0.9–0.9)	0.733
3 months	0.1 (− 0.8–0.8)	0.700	− 0.1 (− 1.0–1.0)	0.767
6 months	0.0 (− 0.6–0.8)	0.291	0.0 (− 0.6–1.0)	0.367
Upper-arm circumference, cm				
Baseline	16.0 (15.0–17.0)	-	16.0 (15.0–17.0)	-
1 month	16.0 (15.0–17.0)	0.026	15.5 (15.0–17.0)	0.544
3 months	16.0 (15.0–18.0)	< 0.001	16.0 (15.0–17.0)	0.029
6 months	17.0 (15.0–18.0)	< 0.001	16.0 (15.0–18.0)	< 0.001^*^
BMI, kg/m^2^				
Baseline	16.3 (15.4–17.7)	-	16.1 (14.9–17.3)	-
1 month	16.5 (15.3–17.8)	0.561	16.1 (15.0–17.3)	0.526
3 months	16.1 (15.0–17.7)	0.494	16.1 (14.8–17.5)	0.964
6 months	16.3 (15.6–17.2)	0.938	15.6 (14.7–17.1)	0.036
Chest circumference, cm				
Baseline	50.5 (47.0–53.5)	-	50.0 (47.0–53.9)	-
1 month	51.0 (47.0–54.5)	0.001	51.0 (47.0–53.9)	0.346
3 months	51.5 (47.5–54.0)	< 0.001	52.0 (47.0–54.0)	0.032
6 months	52.0 (49.0–55.0)	< 0.001	52.0 (49.0–54.0)	< 0.001^*^
Head circumference, cm				
Baseline	48.3 (45.0–50.0)	-	47.7 (46.0–49.5)	-
1 month	48.0 (45.5–49.5)	0.109	47.8 (45.1–49.5)	0.923
3 months	48.5 (46.0–5.0)	< 0.001	48.5 (46.0–50.0)	0.003^*^
6 months	49.0 (47.0–50.0)	< 0.001	49.0 (46.6–50.0)	< 0.001^*^
Height, cm				
Baseline	93.0 (79.0–104.0)	-	92.0 (77.3–100.4)	-
1 month	94.8 (81.0–107.0)	< 0.001	94.3 (79.3–101.9)	< 0.001^*^
3 months	97.0 (82.0–108.0)	< 0.001	95.8 (81.3–104.0)	< 0.001^*^
6 months	98.5 (81.0–109.0)	< 0.001	98.0 (84.4–107.9)	< 0.001^*^
Weight, kg				
Baseline	14.0 (10.0–17.0)	-	13.5 (9.6–16.7)	-
1 month	14.6 (10.2–17.5)	< 0.001	13.4 (10.0–16.8)	0.005^*^
3 months	14.7 (11.2–18.1)	< 0.001	14.3 (10.7–17.2)	< 0.001^*^
6 months	15.0 (11.6–19.2)	< 0.001	15.0 (11.2–18.2)	< 0.001^*^

*Abbreviations*: *IQR* Interquartile range, *SOS* Speed of sound, *BMI* Body mass index

^*^ means significant difference (*p* < 0.017)

**Fig. 3:**
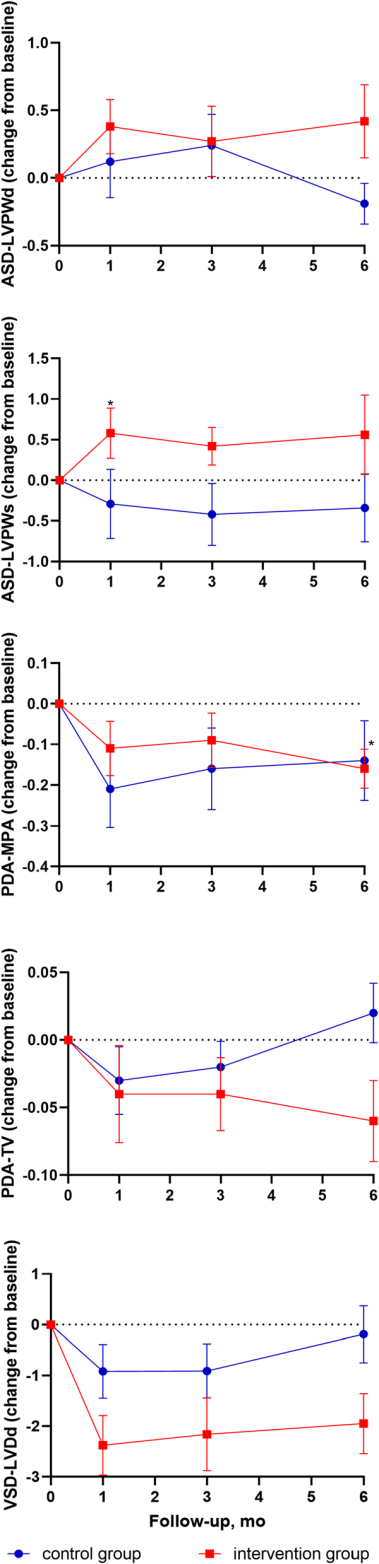
Exercise-related echocardiographic changes by the CHD subtypes. The *X*-axis shows the time points (months) of the follow-up, with “0” representing the preoperative assessment. The *Y*-axis shows the mean changes in the values of the cardiac ultrasound indexes, as calculated by subtracting each of the preoperative values from the postoperative assessment values. The echocardiographic trends differed across the studied CHD subtypes. ASD indicates atrial septal defect. LVDd indicates left ventricular end diastolic dimension. LVPWd indicates left ventricular posterior wall depth. LVPWs indicates left ventricular posterior wall thickness at end-systole. MPA indicates the main pulmonary artery diameter. PDA indicates patent ductus arteriosus. TV indicates tricuspid blood flow velocity. VSD indicates ventricular septal defect. * indicates that the parameters were significantly different between groups at the current assessment (*p* < 0.017)

Exercise had a slight effect when pooled across both treatment groups and all postcatheterization assessments compared with the precatheterization assessment (Additional file 1: Table S2). In a stratified analysis of different CHD subtypes, however, echocardiographic profiles varied significantly between the two groups (Fig. 3; Additional file 1: Fig. S1&2, Table S3a-d). The intervention group had a significantly higher left ventricular posterior wall thickness at end-systole (LVPWs) than the control group with ASD but a significantly lower left ventricular end diastolic dimension (LVDd) than the control group with VSD (Table 5). There were no significant differences in the modified Ross score between the groups or between pre- and postcatherization (Additional file 1: Table S4).

**Table 5:** Mixed-model analyses of echocardiography in CHD

	*F* value	95% CI	*p* value
CHD			
LVDd	0.04	− 1.16–1.24	0.945
LVDs	0.03	− 0.83–0.89	0.941
LVPWd	0.08	− 0.14–0.29	0.484
LVPWs	0.23	− 0.10–0.56	0.175
LVEF	0.44	− 0.49–1.38	0.348
MPA	− 0.04	− 0.23–0.15	0.657
MV	0.00	− 0.02–0.02	0.851
TV	0.01	− 0.02–0.01	0.516
ASD			
LVDd	0.89	− 0.42–2.21	0.176
LVDs	0.59	− 0.48–1.65	0.271
LVPWd	0.27	− 0.08–0.63	0.126
LVPWs	0.74	0.16–1.32	0.014*
LVEF	0.31	− 2.11–2.73	0.795
MPA	− 0.02	− 0.36–0.32	0.918
MV	0.01	− 0.04–0.06	0.678
TV	− 0.00	− 0.05–0.04	0.896
*Z* score of LVDd	0.05	− 0.35–0.45	0.799
*Z* score of LVDs	0.32	− 0.05–0.68	0.086
*Z* score of LVPWd	− 0.03	− 0.40–0.33	0.847
*Z* score of LVPWs	0.12	− 0.34–0.58	0.601
*Z* score of Ao	− 0.26	− 0.86–0.34	0.389
VSD			
LVDd	− 1.16	− 2.23 to − 0.08	0.036*
LVDs	− 0.62	− 1.47–0.22	0.147
LVPWd	− 0.27	− 0.67–0.13	0.182
LVPWs	− 0.52	− 1.09 –0.05	0.073
LVEF	0.05	− 1.27–1.37	0.939
MPA	− 0.02	− 0.10–0.06	0.613
MV	0.00	− 0.04–0.04	0.852
TV	− 0.01	− 0.04–0.03	0.589
*Z* score of LVDd	− 0.22	− 0.48–0.04	0.097
*Z* score of LVDs	− 0.47	− 0.83 to − 0.10	0.012*
*Z* score of LVPWd	− 0.20	− 0.49–0.10	0.183
*Z* score of LVPWs	− 0.74	− 1.19 to − 0.30	0.002*
*Z* score of Ao	0.13	− 0.26–0.53	0.510
PDA			
LVDd	0.00	− 1.50–2.00	0.997
LVDs	− 0.05	− 1.21–1.11	0.935
LVPWd	− 0.07	− 0.37–0.22	0.624
LVPWs	− 0.31	− 0.77–0.15	0.187
LVEF	1.20	− 0.84–3.24	0.242
MPA	− 0.04	− 0.11–0.03	0.285
MV	− 0.02	− 0.06–0.02	0.334
TV	− 0.01	− 0.04–0.02	0.595
*Z* score of LVDd	− 0.13	− 0.43–0.17	0.394
*Z* score of LVDs	0.29	− 0.06–0.64	0.099
*Z* score of LVPWd	− 0.10	− 0.45–0.25	0.558
*Z* score of LVPWs	0.10	− 0.27–0.47	0.601
*Z* score of Ao	0.18	− 0.31–0.66	0.476
PS			
LVDd	− 0.04	− 1.69–1.61	0.965
LVDs	− 0.85	− 2.26–0.56	0.227
LVPWd	− 0.01	− 0.43–0.42	0.980
LVPWs	− 0.44	− 1.35–0.48	0.334
LVEF	1.28	− 1.01–3.58	0.259
MPA	0.25	− 0.24–0.73	0.305
MV	0.01	− 0.05–0.08	0.677
TV	− 0.04	− 0.09–0.02	0.170
*Z* score of LVDd	− 0.32	− 0.84–0.20	0.217
*Z* score of LVDs	− 0.07	− 0.61–0.48	0.803
*Z* score of LVPWd	− 0.41	− 0.98–0.15	0.145
*Z* score of LVPWs	− 0.21	− 1.08–0.66	0.624
*Z* score of Ao	− 0.59	− 1.04 to − 0.14	0.013*

In this table, we compared trends of echocardiography in CHD subtypes separately between the intervention and the control group using mixed effects modeling. *F* values of fixed effects were reported

*ASD* Atrial septal defect, *LVDd* Left ventricular end diastolic dimension, *LVDs* Left ventricular end-systolic dimension, *LVEF* Left ventricular ejection fraction, *LVPWd* Left ventricular posterior wall depth, *LVPWs* Left ventricular posterior wall thickness at end-systole, *MPA* Main pulmonary artery diameter, *MV* Mitral blood flow velocity, *PDA* Patent ductus arteriosus, *PS* Pulmonary stenosis, *TV* Tricuspid blood flow velocity, *VSD* Ventricular septal defect

^*^ means significant difference (*p* < 0.017)

During the 6-month study period, in both treatment groups, we observed significant physical development, including height, weight, and upper-arm, chest, and head circumference (Table 4). Three months postcatheterization, the intervention group had significant increases in upper-arm and chest circumference, whereas physical development was slower in the control group, with significant increases observed at the 6-month assessment. Furthermore, increase was significantly greater in the intervention group as compared to the control group (mixed model: _*fixed effect*_ = 0.35, Table 3).

Parental self-reported anxiety, psychological burden, and quality of life also varied between treatment groups and over time (Additional file 1: Table S6 and Table S5). The intervention group demonstrated significantly higher scores in the quality-of-life assessment at 6 months than in the precatheterization assessment, as measured by the SF-36 total score (median (IQR) 678.0 (611.5–733.5) *p* = 0.001; Additional file 1: Table S5). This was also the case for both the physical (median (IQR) 359.0 (328.0–383.0); *p* = 0.002) and mental (median (IQR) 326.0 (287.5–349.5); *p* = 0.002) component scores (Additional file 1: Table S5). Overall, the postcatheterization SF-36 scores at 6 months were higher in the intervention group than in the control group (Additional file 1: Table S5); in addition, parents in the intervention group had significantly increased quality of life when compared to the those in the control group (mixed model: _*fixed effect*_ = 33.2, 95% CI 6.25–60.15; Table 6).

**Table 6:** Mixed-model analysis of SAS, ZCBS and SF-36 between the intervention and the control groups (*n* = 192)

	*F* value	95% CI	*p* value
SAS score			
SAS rude score	− 0.76	− 1.81–0.30	0.159
SAS standard score	− 0.94	− 2.26–0.37	0.159
ZCBS score	− 1.01	− 3.38–1.37	0.404
SF-36 score			
PCS	14.79	2.10–27.48	0.023
MCS	18.49	2.83–34.16	0.021
Total score	33.20	6.25–60.15	0.016^*^

*MCS* Mental component summary, *PCS* Physical component summary, *SAS* Self-Rating Anxiety Scale, *SF-36* Short Form 36-item Health Survey, *ZCBS* Zarit Caregiver Burden Scale. *F* values of fixed effects were reported

^*^means significant difference (*p* < 0.017)

Although the majority of study participants were from regions outside Shanghai (Additional file 1: Table S1), there were no significant differences in the precatheterization assessment findings among the CHD patients living in different regions (Additional file 1: Table S6). At the end of the study, we did not observe any significant differences in any outcomes between the Shanghai and non-Shanghai residents in the intervention group or the control group (Additional file 1: Table S7).

## Discussion

This study is the first prospective randomized controlled trial to investigate the effects of a home-based exercise training program on the development of children with CHD following interventional cardiac catheterization. Our results demonstrated that after a supervised and customized home-based exercise program, children with CHD had significant improvement in their motor abilities as early as 1 month postcatheterization. The home-based exercise program also led to various direct improvements in cardiac structure, as assessed by echocardiography, for different CHD subtypes [37].

Grey matter in different areas of the brain is responsible for important functions, such as controlling muscle movement, sensory experiences, thinking and feeling, memory and speech, and greater volumes were found to be associated with better performance on a task-switching paradigm [38]. Smaller grey matter volumes were reported in toddlers with CHD before cardiac surgery, which may be related to impaired grey matter growth due to poor cerebral oxygen delivery [39]. Moreover, due to abnormal hemodynamics and hypoxia during gestation, neurodevelopmental delays and behavioral impairments are common in children with CHD [4, 12, 40]. Early surgical repair of the heart defect alone cannot prevent the onset of motor impairments [21, 41]. Therefore, an early empirical study suggested that postoperative (postcatheterization) exercise might compensate for neurodevelopmental delay [42] and induce adaptive changes in the cardiovascular system and natural cardiac remodeling [37, 43].

It has been shown that physical activity could promote brain remodeling by regulating epigenetics, neuroplasticity, and neurotrophins in animal models [44], and levels of physical activity, which could improve oxygen delivery to the brain, have been positively associated with several structural properties of grey matter [45]. Our results are congruent with those of Stieber et al., who found an increase in the rate of motor development to age-appropriate levels in children with CHD between 12 and 26 months of age with completion of a postoperative home-based rehabilitation program [46]. Our study expanded on this evidence base by including a larger sample size with older children and assessments at 4 different time points, including 3 postcatheterization assessments over 6 months in a randomized controlled trial.

No significant difference in bone strength was found in this study, although previous systematic reviews reported low- to moderate-quality evidence and suggested that increased or higher physical activity levels were positively associated with bone and skeletal health in the early years (aged 0–6 years) [47]. Given the absence of this information in the extant literature, there is a clear need to design experimental trials and prospective cohort studies to answer the question of whether a dose–response relationship exists between physical activity and health during this early period of the lifespan and, if so, what the nature of the relationship is.

Significant changes in the modified Ross scores for heart failure were not observed in this study, although different cardiac changes on echocardiography associated with specific CHD subtypes were demonstrated, including ASD, VSD, and PDA. Specifically, children with ASD have left-to-right shunts at the atrial level, causing excessive motion of the right ventricle with right ventricular volume overloa [48]. As a result, these children have increased blood volume in the left and right atrium, as well as increased right ventricular stroke volume. We found that the home-based exercise program could significantly reduce the velocity of tricuspid flow (TV), which would be beneficial to reducing right ventricular volume overload in children with ASD. Compared to children with ASD, children with VSD have left-to-right shunts at the ventricular level, causing increased pulmonary artery pressure and left atrial volume [49]. Echocardiographic observations suggest that training parents in a customized home-based exercise program, delivered under supervision, could help reduce the inner diameters of the left ventricular and pulmonary arteries and improve cardiac structure in children with VSD. For children with PDA, echocardiographic assessments also suggested that family exercise training could help reduce the left ventricle diameter and facilitate cardiac structural remodeling. Therefore, family exercise training might facilitate the improvement of cardiac structure in children with CHD.

With a CHD diagnosis in their children, the quality of life of parents is often impacted by anxiety caused by procedures and treatments, financial pressures relating to medical care, and the burden of caring for their children [50, 51]. These tensions can also affect their children’s health [52], suggesting that a family-focused postoperative treatment plan would potentially benefit both children with CHD and their parents. A randomized controlled trial of 56 families with children with CHD showed that four 90-min sessions of educational programs over 4 weeks increased the parents’ quality of life, as well as their self-efficacy, as assessed by the quality of life questionnaire (SF-36) and Generalized Self-Efficacy (GSE) scale [53]. Consistently, our findings demonstrate that intensive training and customized and supervised home-based exercise can effectively improve parents’ quality of life [53]. Moreover, with continuous support and professional instruction from study administrators, children with CHD and their parents performed exercises together and interacted more closely and frequently, which likely helped to foster better family relations and reduced the emotional pressure that the parents experienced. The improved motor development after exercise training in both the control and intervention groups likely also contributed to improved quality of life for the parents.

In this study, participating families who did not live locally and only traveled to Shanghai for catheterization and postcatheterization assessments, and daily rehabilitation exercises during the 6-month study period were completed under the remote guidance and supervision of the study team in Shanghai via WeChat and phone calls. It is likely that the multidisciplinary team approach in designing a safe, customized exercise program with close monitoring via telecommunication technologies was critical for achieving the high completion rate in this study, irrespective of distance to the studied hospital. Completing the program led to significant improvements regarding postoperative rehabilitation in children with CHD, as the largest changes were observed after 6 months. In addition, the nonsignificant difference between the results from Shanghai and rural areas provided strong evidence that a tertiary hospital can provide effective and low-cost rehabilitation services to a very large patient population via telehealth technologies. The use of such technologies also enabled families with children with CHD to save time and travel costs.

Our study was also limited by the use of telehealth technologies, such as the WeChat app, which presented challenges, particularly for participants living in rural areas. Occasional technical glitches discouraged some patients from participating, with several leaving the study (*n* = 7). Additionally, establishing physical activity levels from questionnaires remains challenging and is not an ideal estimate of physical fitness. After observing the overwhelmingly positive results in the 6-month assessment, we terminated this study earlier than originally planned, and therefore, we do not know whether the observed motor symptoms persisted beyond the intervention [28]. For future studies, we recommend large-scale, multicenter controlled investigations of children with CHD with longer follow-up periods to evaluate the long-term effects of home-based exercise. We also recommend more communication between parents and doctors and therapists to improve patients’ motivation to exercise and thus improve their motor functioning and physical performance.

## Conclusions

In conclusion, the trial provides evidence that parental training in customized and supervised home-based exercise significantly increases motor quotient scores in CHD patients within 6 months of catheterization. This study therefore contributes to the evidence for the benefits of exercise among pediatric simple CHD patients. Furthermore, the high retention rate of our study, which used technological innovations to engage patients remotely, suggests a viable method for effective intervention for further studies and for patients requiring intervention during pandemic conditions. Future studies will monitor the progression of CHD within this cohort, and we are optimistic that, with continued supervision and guidance, these children with CHD will match their peers in terms of development.

### Supplementary Information

**Additional file 1.** Randomization and masking. Protocol modifications. **Figure S1.** Post-catheterization echocardiographic changes by the CHD subtypes in the intervention group. **Figure S2.** Post-catheterization echocardiographic changes by the CHD subtypes in the control group. **Table S1.** Outlines of the home-based exercise program differed by developmental age. **Table S2.** The Wilcoxon rank sum test of secondary outcomes (echocardiography) for congenital heart disease (CHD) against preoperative assessment. **Table S3.** The Wilcoxon rank sum test of secondary outcomes (echocardiography) against preoperative assessment in CHD subtypes. **Table S3a.** The Wilcoxon rank sum test of secondary outcomes (echocardiography) for atrial septal defect (ASD) against preoperative assessment. **Table S3b.** The Wilcoxon rank sum test of secondary outcomes (echocardiography) for ventricular septal defect (VSD) against preoperative assessment. **Table S3c.** The Wilcoxon rank sum test of secondary outcomes (echocardiography) for patent ductus arteriosus (PDA) against preoperative assessment. **Table S3d.** The Wilcoxon rank sum test of secondary outcomes (echocardiography)y for pulmonary stenosis (PS) against preoperative assessment. **Table S4.** The Wilcoxon rank sum test of secondary outcomes (modified Ross score) against preoperative assessment. **Table S5.** The Wilcoxon rank sum test of secondary outcomes (parents’ anxiety, burden and quality of life) against preoperative assessment. **Table S6.** Characteristics of the enrolled CHD children with various residencies at baseline (*n* = 192). **Table S7.** Outcomes of the CHD children with various residencies in the intervention group and the control group separately. **Table S7a.** Outcomes of the CHD children with various residencies in the control group at 6 months after catheterization (*n* = 97). **Table S7b.** Outcomes of the CHD children with various residencies in the intervention group at 6 months after catheterization (*n* = 95).

## Data Availability

The authors affirm that the manuscript (including the additional file) is an honest, accurate, and transparent account of the study being reported; that no important aspects of the study have been omitted; and that any discrepancies are disclosed. The data used in this study will be made available to scientific researchers upon approval of their study protocol and analysis plan by study team committee. Proposals should be directed to the corresponding author and the first author. A data sharing agreement will need to be signed by data requestors, and after obtaining the approval of the Xinhua Hospital Ethics Committee for the project, the data will be released.

## Abbreviations

AEs: Adverse events
ASD: Atrial septal defect
BMI: Body mass index
CHD: Congenital heart disease
FMQ: Fine motor quotient
GMQ: Gross motor quotient
IQRs: Interquartile ranges
ITT: Intention-to-treat
LVPWs: Left ventricular posterior wall thickness at end-systole
LVDd: Left ventricular end diastolic dimension
PDA: Patent ductus arteriosus
PS: Pulmonary stenosis
SOS: Speed of sound
SAS: Self-rating anxiety scale
SF-36: 36-Item short-form health survey
TV: Tricuspid blood flow velocity
TMQ: Total motor quotient
VSD: Ventricular septal defect
ZCBS: Zarit caregiver burden scale

## References

[1] Liu Y, Chen S, Zühlke L (2019). Global birth prevalence of congenital heart defects 1970–2017: updated systematic review and meta-analysis of 260 studies. Int J Epidemiol.

[2] Mandalenakis Z, Giang KW, Eriksson P (2020). Survival in children with congenital heart disease: have we reached a peak at 97%?. J Am Heart Assoc.

[3] Mahle WT, Spray TL, Wernovsky G (2000). Survival after reconstructive surgery for hypoplastic left heart syndrome: a 15-year experience from a single institution. Circulation.

[4] Marino BS, Lipkin PH, Newburger JW (2012). Neurodevelopmental outcomes in children with congenital heart disease: evaluation and management: a scientific statement from the American Heart Association. Circulation.

[5] Razzaghi H, Oster M, Reefhuis J (2015). Long-term outcomes in children with congenital heart disease: National Health Interview Survey. J Pediatr.

[6] Dallaire F, Sarkola T (2018). Growth of cardiovascular structures from the fetus to the young adult. Adv Exp Med Biol.

[7] Okely AD, Ghersi D, Hesketh KD (2017). A collaborative approach to adopting/adapting guidelines - The Australian 24-Hour Movement Guidelines for the early years (Birth to 5 years): an integration of physical activity, sedentary behavior, and sleep. BMC Public Health.

[8] WHO Guidelines Approved by the Guidelines Review Committee (2019). Guidelines on Physical Activity, Sedentary Behaviour and Sleep for Children under 5 Years of Age.

[9] Guan H, Zhang Z, Wang B (2020). Proportion of kindergarten children meeting the WHO guidelines on physical activity, sedentary behaviour and sleep and associations with adiposity in urban Beijing. BMC Pediatr.

[10] Bjarnason-Wehrens B, Dordel S, Schickendantz S (2007). Motor development in children with congenital cardiac diseases compared to their healthy peers. Cardiol Young.

[11] Longmuir PE, McCrindle BW (2009). Physical activity restrictions for children after the Fontan operation: disagreement between parent, cardiologist, and medical record reports. Am Heart J.

[12] Calderon J, Bellinger DC (2015). Executive function deficits in congenital heart disease: why is intervention important?. Cardiol Young.

[13] Fourdain S, Simard MN, Dagenais L, et al. Gross motor development of children with congenital heart disease receiving early systematic surveillance and individualized intervention: brief report. Dev Neurorehabil. 2020: 1–7. 10.1080/17518423.2020.1711541. 10.1080/17518423.2020.1711541 31928274

[14] Cohen MS (2012). Clinical practice: the effect of obesity in children with congenital heart disease. Eur J Pediatr.

[15] Siaplaouras J, Niessner C, Helm PC (2020). Physical activity among children with congenital heart defects in Germany: a nationwide survey. Front Pediatr.

[16] Kolt GS, Ferdman BR, Choi JY (2020). Emotional quality-of-life and patient-reported limitation in sports participation in children with uncorrected congenital and acquired heart disease in healthcare-restricted settings in low- and middle-income countries. Cardiol Young.

[17] Yang X, Sun K, Du Q (2015). Development of motor cognition and language in children with congenital heart disease. Chin J Appl Clin Pediatr.

[18] Fletcher GF, Landolfo C, Niebauer J (2018). Promoting physical activity and exercise: JACC Health Promotion Series. J Am Coll Cardiol.

[19] Quist JS, Winther J, Friis AL (2022). Maintenance of cardiorespiratory fitness, body composition, and a physically active lifestyle after structured exercise interventions in individuals with overweight and obesity: a mixed-method follow-up study. Public Health Pract.

[20] Nieman DC, Sakaguchi CA (2022). Physical activity lowers the risk for acute respiratory infections: time for recognition. J Sport Health Sci.

[21] Majnemer A, Limperopoulos C, Shevell MI (2009). A new look at outcomes of infants with congenital heart disease. Pediatr Neurol.

[22] Guessoum SB, Lachal J, Radjack R (2020). Adolescent psychiatric disorders during the COVID-19 pandemic and lockdown. Psychiatry Res.

[23] Meyer M, Brudy L (2020). Current state of home-based exercise interventions in patients with congenital heart disease: a systematic review. Heart.

[24] Bravo-Escobar R, Gonzalez-Represas A, Gomez-Gonzalez AM (2017). Effectiveness and safety of a home-based cardiac rehabilitation programme of mixed surveillance in patients with ischemic heart disease at moderate cardiovascular risk: a randomized, controlled clinical trial. BMC Cardiovasc Disord.

[25] Kraal JJ, Van den Akker-Van Marle ME, Abu-Hanna A (2017). Clinical and cost-effectiveness of home-based cardiac rehabilitation compared to conventional, centre-based cardiac rehabilitation: Results of the FIT@Home study. Eur J Prev Cardiol.

[26] Amir NH, Dorobantu DM, Wadey CA (2022). Exercise training in paediatric congenital heart disease: fit for purpose?. Arch Dis Child.

[27] Zhou X, Du Q, Sun K (2017). GW28-e0722 Randomized controlled trial of a home-based exercise program for children with congenital heart disease following interventional cardiac catheterization: a preliminary study. J Am Coll Cardiol.

[28] Du Q, Salem Y, Liu HH (2017). A home-based exercise program for children with congenital heart disease following interventional cardiac catheterization: study protocol for a randomized controlled trial. Trials.

[29] Warnes CA, Liberthson R, Danielson GK (2001). Task force 1: the changing profile of congenital heart disease in adult life. J Am Coll Cardiol.

[30] Ross RD (2012). The Ross classification for heart failure in children after 25 years: a review and an age-stratified revision. Pediatr Cardiol.

[31] Mitting R, Marino L, Macrae D (2015). Nutritional status and clinical outcome in postterm neonates undergoing surgery for congenital heart disease. Pediatr Crit Care Med.

[32] Zung WW (1971). A rating instrument for anxiety disorders. Psychosomatics.

[33] Ankri J, Andrieu S, Beaufils B (2005). Beyond the global score of the Zarit Burden Interview: useful dimensions for clinicians. Int J Geriatr Psychiatry.

[34] Zarit SH, Reever KE, Bach-Peterson J (1980). Relatives of the impaired elderly: correlates of feelings of burden. Gerontologist.

[35] Liu JF, Xie WP, Lei YQ, et al. The relationship between religious beliefs and mental state, care burden, and quality of life in parents of infant patients with congenital heart disease. Cardiol Young. 2021: 1–5. 10.1017/s1047951121004200. 10.1017/S1047951121004200 34645537

[36] Khan KM, Sarafoglou K, Somani A (2013). Can ultrasound be used to estimate bone mineral density in children with growth problems?. Acta Paediatr.

[37] Pascotto M, Santoro G, Cerrato F (2006). Time-course of cardiac remodelling following transcatheter closure of atrial septal defect. Int J Cardiol.

[38] Erickson KI, Leckie RL, Weinstein AM (2014). Physical activity, fitness, and gray matter volume. Neurobiol Aging.

[39] Bonthrone AF, Dimitrova R, Chew A (2021). Individualized brain development and cognitive outcome in infants with congenital heart disease. Brain Commun.

[40] Khalil A, Suff N, Thilaganathan B (2014). Brain abnormalities and neurodevelopmental delay in congenital heart disease: systematic review and meta-analysis. Ultrasound Obstet Gynecol.

[41] Brosig CL, Bear L, Allen S (2017). Preschool neurodevelopmental outcomes in children with congenital heart disease. J Pediatr.

[42] Longmuir PE, Tremblay MS, Goode RC (1990). Postoperative exercise training develops normal levels of physical activity in a group of children following cardiac surgery. Pediatr Cardiol.

[43] Helal L, Silveira AD (2018). The nature of cardiac remodelling due to physical exercise: more evidence towards to the normal adaptive responses of the heart. Arq Bras Cardiol.

[44] Liang J, Wang H, Zeng Y (2021). Physical exercise promotes brain remodelling by regulating epigenetics, neuroplasticity and neurotrophins. Rev Neurosci.

[45] Erickson KI, Voss MW, Prakash RS (2011). Exercise training increases size of hippocampus and improves memory. Proc Natl Acad Sci U S A.

[46] Stieber NA, Gilmour S, Morra A (2012). Feasibility of improving the motor development of toddlers with congenital heart defects using a home-based intervention. Pediatr Cardiol.

[47] Pate RR, Hillman CH, Janz KF (2019). Physical activity and health in children younger than 6 years: a systematic review. Med Sci Sports Exerc.

[48] Hatani Y, Tanaka H, Mochizuki Y (2018). Left ventricular dispersion as a parameter for augmented left ventricular stroke volume in patients with atrial septal defect following transcatheter closure. Echocardiography (Mount Kisco, NY).

[49] Karonis T, Scognamiglio G, Babu-Narayan SV (2016). Clinical course and potential complications of small ventricular septal defects in adulthood: late development of left ventricular dysfunction justifies lifelong care. Int J Cardiol.

[50] Sileshi L, Tefera E (2017). Health-related quality of life of mothers of children with congenital heart disease in a sub-Saharan setting: cross-sectional comparative study. BMC Res Notes.

[51] Lawoko S, Soares JJF (2006). Psychosocial morbidity among parents of children with congenital heart disease: a prospective longitudinal study. Heart Lung.

[52] Kim Y, Baker F, Spillers RL (2007). Cancer caregivers’ quality of life: effects of gender, relationship, and appraisal. J Pain Symptom Manag.

[53] Edraki M, Kamali M, Beheshtipour N (2014). The effect of educational program on the quality of life and self-efficacy of the mothers of the infants with congenital heart disease: a randomized controlled trial. Int J Comm Based Nurs Midwifery.

