# Supplementary material for: Effects of 6-month customized home-based exercise on motor development, bone strength, and parental stress in children with simple congenital heart disease: a single-blinded randomized clinical trial
Source: BMC Med. 2024 Feb 6;22:27. doi: 10.1186/s12916-023-03242-6 (PMC10845703; doi:10.1186/s12916-023-03242-6)
Supplement: Supplementary file 1 — Additional file 1. Randomization and masking. Protocol modifications. Figure S1. Post-catheterization echocardiographic changes by the CHD subtypes in the intervention group. Figure S2. Post-catheterization echocardiographic changes by the CHD subtypes in the control group. Table S1. Outlines of the home-based exercise program differed by developmental age. Table S2. The Wilcoxon rank sum test of secondary outcomes (echocardiography) for congenital heart disease (CHD) against preoperative assessment. Table S3. The Wilcoxon rank sum test of secondary outcomes (echocardiography) against preoperative assessment in CHD subtypes. Table S3a. The Wilcoxon rank sum test of secondary outcomes (echocardiography) for atrial septal defect (ASD) against preoperative assessment. Table S3b. The Wilcoxon rank sum test of secondary outcomes (echocardiography) for ventricular septal defect (VSD) against preoperative assessment. Table S3c. The Wilcoxon rank sum test of secondary outcomes (echocardiography) for patent ductus arteriosus (PDA) against preoperative assessment. Table S3d. The Wilcoxon rank sum test of secondary outcomes (echocardiography)y for pulmonary stenosis (PS) against preoperative assessment. Table S4. The Wilcoxon rank sum test of secondary outcomes (modified Ross score) against preoperative assessment. Table S5. The Wilcoxon rank sum test of secondary outcomes (parents’ anxiety, burden and quality of life) against preoperative assessment. Table S6. Characteristics of the enrolled CHD children with various residencies at baseline (n = 192). Table S7. Outcomes of the CHD children with various residencies in the intervention group and the control group separately. Table S7a. Outcomes of the CHD children with various residencies in the control group at 6 months after catheterization (n = 97). Table S7b. Outcomes of the CHD children with various residencies in the intervention group at 6 months after catheterization (n = 95). [file 12916_2023_3242_MOESM1_ESM.docx]

**Additional File 1**

**Effects of 6-Month Customized Home-based Exercise on Motor Development, Bone Strength and Parental Stress in Children with Simple Congenital Heart Disease: A Single-blinded Randomized Clinical Trial**

[Randomization and masking 3](#_Toc26005)

[Protocol modifications 4](#_Toc7078)

[Supplementary Figures 5](#_Toc27701)

[Figure S1. Post-catheterization echocardiographic changes by the CHD subtypes in the intervention group 6](#_Toc14133)

[Figure S2. Post-catheterization echocardiographic changes by the CHD subtypes in the control group 8](#_Toc8537)

[Supplementary Tables 10](#_Toc15858)

[Table S1 Outlines of the home-based exercise program differed by developmental age. 11](#_Toc25037)

[Table S2. The Wilcoxon rank sum test of secondary outcomes (echocardiography) for congenital heart disease (CHD) against preoperative assessment 14](#_Toc10550)

[Table S3. The Wilcoxon rank sum test of secondary outcomes (echocardiography) against preoperative assessment in CHD subtypes 15](#_Toc6716)

[Table S3a. The Wilcoxon rank sum test of secondary outcomes (echocardiography) for atrial septal defect (ASD) against preoperative assessment 16](#_Toc4312)

[Table S3b. The Wilcoxon rank sum test of secondary outcomes (echocardiography) for ventricular septal defect (VSD) against preoperative assessment 18](#_Toc12716)

[Table S3c. The Wilcoxon rank sum test of secondary outcomes (echocardiography) for patent ductus arteriosis (PDA) against preoperative assessment 20](#_Toc23587)

[Table S3d. The Wilcoxon rank sum test of secondary outcomes (echocardiography)y for pulmonary stenosis (PS) against preoperative assessment 22](#_Toc9495)

[Table S4. The Wilcoxon rank sum test of secondary outcomes (modified Ross score) against preoperative assessment 25](#_Toc25501)

[Table S5. The Wilcoxon rank sum test of secondary outcomes (parents’ anxiety, burden and quality of life) against preoperative assessment 26](#_Toc17557)

[Table S6. Characteristics of the enrolled CHD children with various residencies at baseline (n=192) 29](#_Toc20996)

[Table S7. Outcomes of the CHD children with various residencies in the intervention group and the control group separately 31](#_Toc18150)

[Table S7a. Outcomes of the CHD children with various residencies in the control group at 6 months after catheterization (n=97) 32](#_Toc17956)

[Table S7b. Outcomes of the CHD children with various residencies in the intervention group at 6 months after catheterization (n=95) 33](#_Toc20076)

# Randomization and masking

After the parents signed the written informed consent, a study administrator assigned each participant to either the intervention group or the control group, according to computer-generated random numbers. The allocations were placed in sequentially numbered, sealed envelopes with a signature across the seal. A trained research assistant, blinded to the allocation, enrolled patients and assigned them according to study protocol.

# Protocol modifications

The original protocol for safety supervision of home-based exercise training suggested the use of a portable device so that parents could measure the blood oxygen saturation and heart rates of children with CHD.^1^ Initially, we provided a fingertip pulse oximeter. However, some parents informed us of malfunctions caused by insufficient contact between child’s finger and device, while others reported that device use disrupted the exercises. Thus, with the approval from the Xinhua Hospital Ethics Committee, we stopped use of the Fingertip Pulse Oximeter, and instead we taught parents to monitor their children’s complexion, sweating, and heart rate (a targeted range of 60–80% of maximum heart rate).

The results of a pilot study showed that the motor quotients of CHD subtypes, including PDA, PS, VSD, and ASD had different effect size, with 1.06, 1.70, 0.55, 1.59 respectively. Thus, with study potential attritions setting at α=0.05 and β=0.1, this study should recruit 54 PDA cases, 26 PS cases, 192 VSD cases, and 28 ASD cases, with a total sample size of 300 CHD patients. When 192 subjects finished the 6-months follow-up, we performed an interim analysis. Considering the clinical outcome changes of these subjects, observed by the statistically significant difference of GMQ, FMQ and TMQ between the control group and intervention group at 6 months after catheterization of more than 98% (99.88%, 98.92%, 99.95%), we decided to terminate the study with the approval from the Xinhua Hospital Ethics Committee.

Reference:

1. Du Q, Salem Y, Liu HH, et al. A home-based exercise program for children with congenital heart disease following interventional cardiac catheterization: study protocol for a randomized controlled trial. Trials 2017;18:38-.

# Supplementary Figures

##
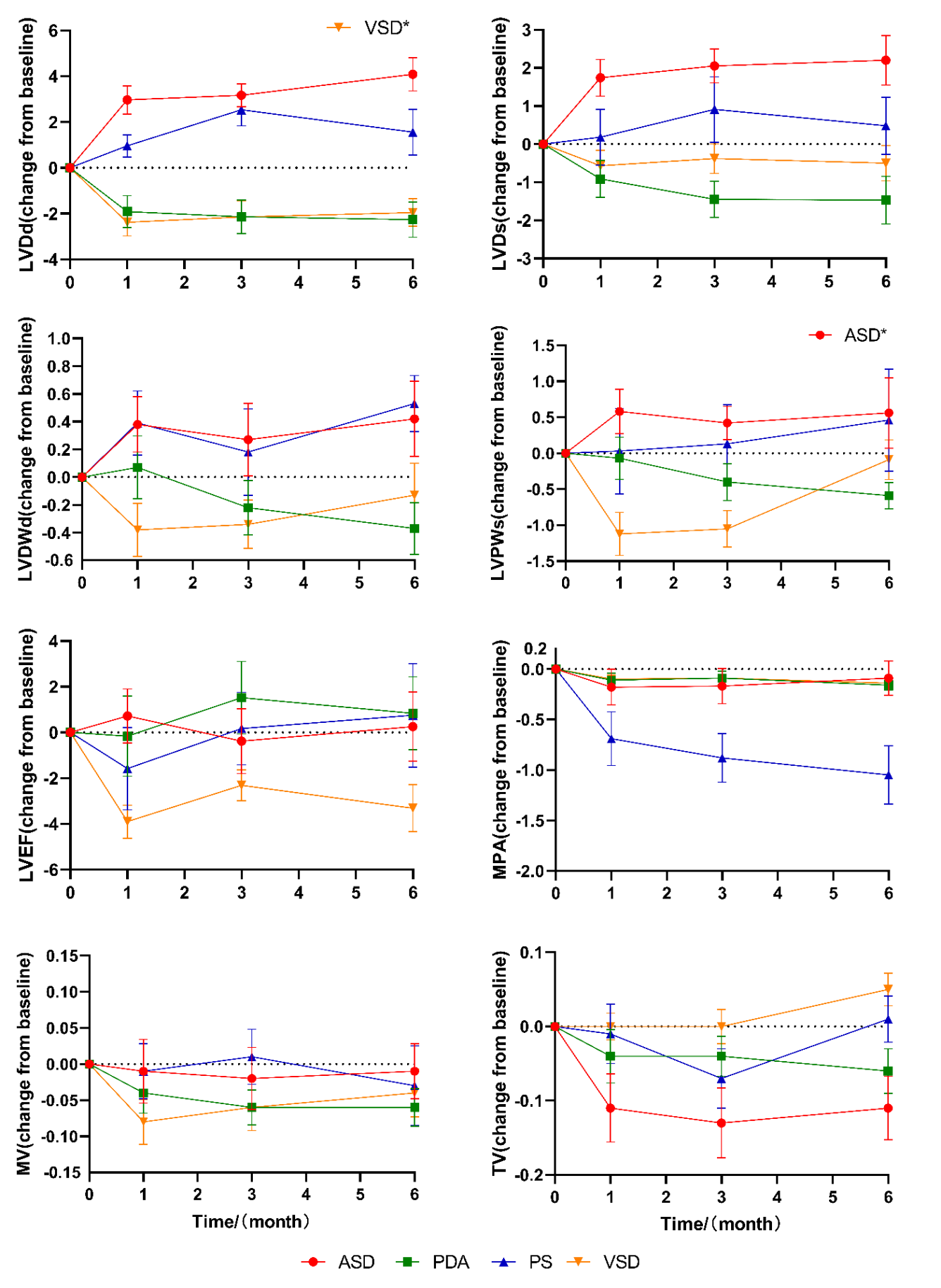
Figure S1. Post-catheterization echocardiographic changes by the CHD subtypes in the intervention group

The X-axis shows the timepoints (months) of the assessments, with “0” representing the preoperative assessment. The Y-axis shows the mean changes in the values of the cardiac ultrasound indexes, as calculated by subtracting each of the preoperative values from the postoperative assessment values, in the intervention group.

For VSD patients, comparing with the preoperative assessment, the intervention group showed significant changes in LVDd at 6-month assessment. For ASD patients, comparing with the preoperative assessment, the intervention group showed significant changes in LVPWs at 6-month assessment.

ASD: atrial septal defect; LVDd: left ventricular end diastolic dimension; LVDs: left ventricular end-systolic dimension; LVEF: left ventricular ejection fraction; LVPWd: left ventricular posterior wall depth; LVPWs: left ventricular posterior wall thickness at end-systole; MPA: main pulmonary artery diameter; MV: mitral blood flow velocity; PDA: patent ductus arteriosus; PS: pulmonary stenosis; TV: tricuspid blood flow velocity; VSD: ventricular septal.

##
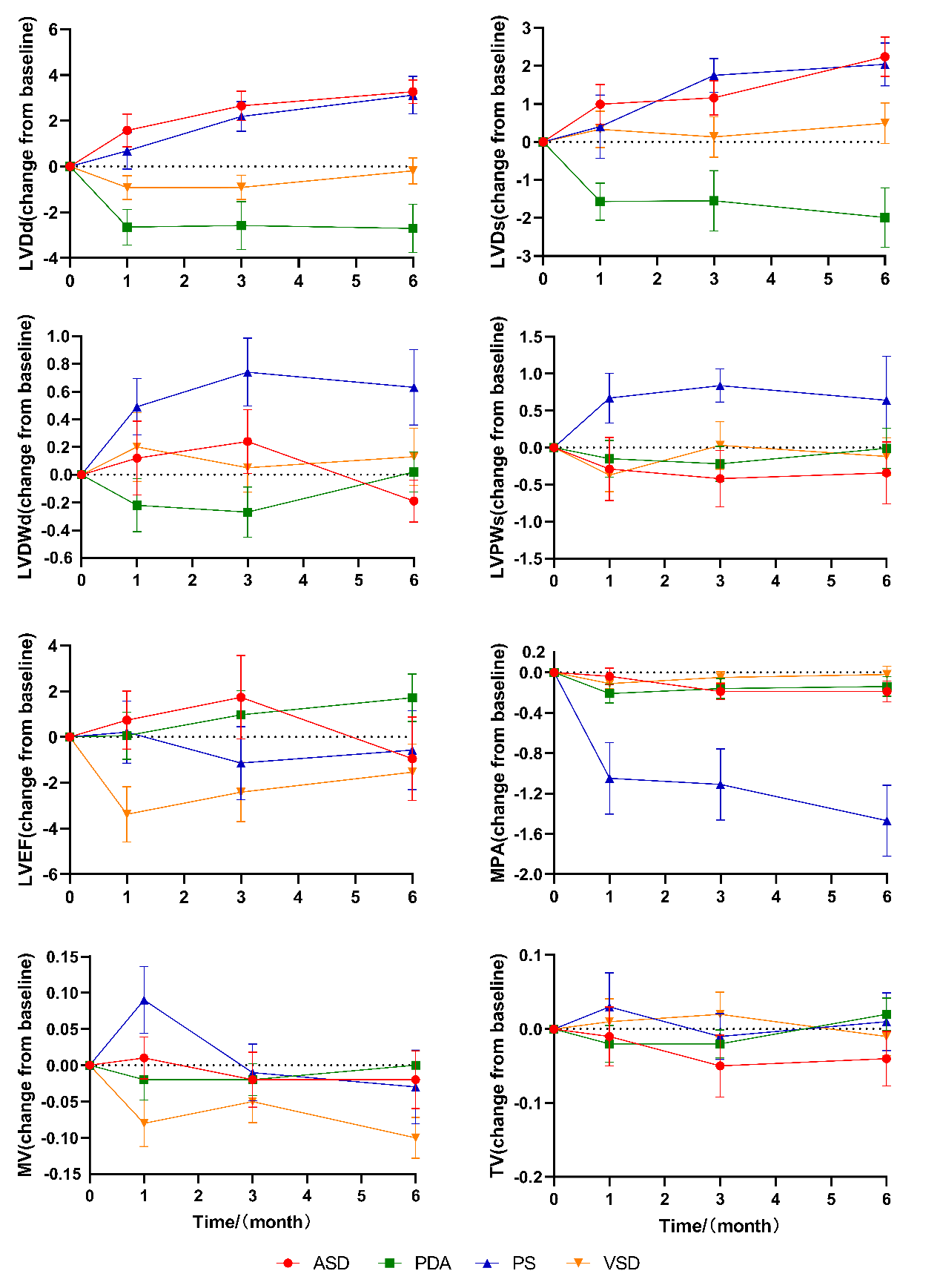
Figure S2. Post-catheterization echocardiographic changes by the CHD subtypes in the control group

The X-axis shows the timepoints (months) of the assessments, with “0” representing the preoperative assessment. The Y-axis shows the mean changes in the values of the cardiac ultrasound indexes, as calculated by subtracting each of the preoperative values from the postoperative assessment values, in the control group. For the four studied CHD subtypes, there were no significant difference in the cardiac ultrasound indexes in the control group.

ASD: atrial septal defect; LVDd: left ventricular end diastolic dimension; LVDs: left ventricular end-systolic dimension; LVEF: left ventricular ejection fraction; LVPWd= left ventricular posterior wall depth; LVPWs: left ventricular posterior wall thickness at end-systole; MPA: main pulmonary artery diameter; MV: mitral blood flow velocity; PDA: patent ductus arteriosus; PS: pulmonary stenosis; TV: tricuspid blood flow velocity; VSD: ventricular septal.

# Supplementary Tables

## Table S1 Outlines of the home-based exercise program differed by developmental age.

| Age | Overview of home-based exercise program* |
| --- | --- |
| - 1. months | - Developmental activities: activities/games with different postures, such as head lifting, support in prone position, hand, or elbow support, etc. For example, the infant could lie on the mother’s leg in a prone position; the mother could shake a sound toy over the infant’s head to induce the infant to lift his/her head or hand; the infant can also lie on a big ball in a prone position. - Passive exercise: stretching the infant’s limbs and shoulder, and wrist and leg manipulation by the parents, such as clapping or nudging the infant’s feet. |
| 7–12 months | - Developmental activities: activities in different positions (prone, sitting, crawling, creeping, kneeling, and standing). - Passive exercise: stretching the infant’s limbs and shoulder, and wrist and leg manipulation by the parents, like the baby’s feet touching the mother’s feet with bending and extending movements, and stepping on a bicycle. |
| 13–24 months | - Postural training: kneeling and standing. - Flexibility training: active stretching of the upper and lower limbs, chest expansion, and shoulder, wrist, and leg movement. - Breathing exercises: abdominal respiration, resisted breathing, deep breathing, and blowing bubbles and pinwheels. - Developmental activities: walking, stair activities, stepping activities, and throwing a ball. - Aerobic endurance training: swimming, riding a bike, and walking. |
| 25–60 months | - Postural training: single-leg standing, standing on tiptoe, single-leg jumping, such as jumping following a rope with snake shapes, rope skipping, or standing on a soft cushion. - Flexibility training: active stretching of the upper and lower limbs, chest expansions, and shoulder, wrist, and leg movements. - Breathing training: abdominal respiration, resistant breathing, deep breathing, and blowing bubbles and pinwheels. - Muscle strength training: pulling elastic bands with the upper limbs, squatting down and standing up, straight-leg raising movements, and gluteus training, like hiding in a big box, and inducing the child out with preferred toys. - Developmental activities: climbing upstairs and coming downstairs, stepping activities, and throwing and kicking a ball. - Aerobic endurance training: swimming, riding a bike, walking, jogging, and running to catch things with a crossing obstacle. |

* Parents implemented the rehabilitation program at home over a 6-month period; the total daily time request will be 30 minutes for no less than 5 days per week.

## Table S2. The Wilcoxon rank sum test of secondary outcomes (echocardiography) for congenital heart disease (CHD) against preoperative assessment

|  | **Intervention group(n=95)** | | **Control group(n=97)** | |
| --- | --- | --- | --- | --- |
|  | **median (IQR)** | ***p* value** | **median (IQR)** | ***p* value** |
| **Left ventricular end diastolic dimension (LVDd)** | | | | |
| Baseline | 33.3 (29.3-36.3) | - | 32.8 (29.9-36.1) | - |
| 1 month | 32.9 (29.5-35.8) | .433 | 32.5 (30.0-34.8) | .611 |
| 3 months | 33.2 (30.0-35.6) | .518 | 33.1 (30.5-36.0) | .946 |
| 6 months | 32.5 (30.3-35.5) | .527 | 33.4 (30.3-36.0) | .602 |
| **Left ventricular end-systolic dimension (LVDs)** | | | | |
| Baseline | 21.1 (18.8-23.1) | - | 20.8 (18.6-23.3) | - |
| 1 month | 21.2 (18.9-22.8) | .903 | 21.0 (18.7-23.2) | .994 |
| 3 months | 21.7 (18.4-23.2) | .950 | 20.6 (18.8-23.3) | .907 |
| 6 months | 20.8 (19.1-23.2) | .840 | 21.3 (19.2-23.5) | .555 |
| **Left ventricular posterior wall depth (LVPWDd)** | | | | |
| Baseline | 4.6 (4.0-5.1) | - | 4.4 (3.8-5.0) | - |
| 1 month | 4.6 (3.8-5.2) | .961 | 4.6 (3.7-5.1) | .916 |
| 3 months | 4.4 (3.8-5.0) | .181 | 4.5 (3.8-5.1) | .587 |
| 6 months | 4.5 (3.7-5.2) | .434 | 4.5 (3.8-5.1) | .628 |
| **Left ventricular posterior wall thickness at end-systole (LVPWs)** | | | | |
| Baseline | 7.6 (7.0-9.0) | - | 7.3 (6.5-8.5) | - |
| 1 month | 7.6 (6.7-8.4) | .323 | 7.1 (6.5-8.2) | .438 |
| 3 months | 7.3 (6.5-8.0) | .046 | 7.3 (6.3-8.2) | .584 |
| 6 months | 7.3 (6.4-8.3) | .097 | 7.2 (6.4-8.4) | .834 |
| **Left ventricular ejection fraction (LVEF)** | | | | |
| Baseline | 69.0 (65.0-71.0) | - | 66.0 (64.0-70.0) | - |
| 1 month | 66.0 (62.0-70.0) | .008* | 66.0 (61.0-70.0) | .185 |
| 3 months | 67.0 (63.0-71.0) | .205 | 66.0 (62.0-70.0) | .482 |
| 6 months | 67.0 (62.5-70.0) | .080 | 67.0 (63.0-70.0) | .771 |
| **Main pulmonary artery diameter (MPA)** | | | | |
| Baseline | 1.2 (1.0-1.4) | - | 1.2(1.0-1.4) | - |
| 1 month | 1.0 (1.0-1.2) | .002* | 1.0 (1.0-1.2) | .064 |
| 3 months | 1.0 (1.0-1.2) | .020 | 1.0 (1.0-1.3) | .118 |
| 6 months | 1.0 (1.0-1.2) | .000* | 1.0 (1.0-1.3) | .236 |
| **Mitral blood flow velocity (MV)** | | | | |
| Baseline | 1.0 (1.0-1.2) | - | 1.0 (1.0-1.2) | - |
| 1 month | 1.0 (1.0-1.1) | .023 | 1.0 (1.0-1.2) | .191 |
| 3 months | 1.0 (1.0-1.0) | .021 | 1.0 (1.0-1.1) | .139 |
| 6 months | 1.0 (1.0-1.0) | .028 | 1.0 (1.0-1.0) | .014* |
| **Tricuspid blood flow velocity (TV)** | | | | |
| Baseline | 0.9 (0.8-1.0) | - | 0.8 (0.8-0.8) | - |
| 1 month | 0.8 (0.8-0.8) | .085 | 0.8 (0.8-0.8) | .947 |
| 3 months | 0.8 (0.8-0.8) | .008* | 0.8 (0.8-0.8) | .707 |
| 6 months | 0.8 (0.8-0.8) | .268 | 0.8 (0.8-0.8) | .845 |

IQR: the interquartile range

* means significant difference(P<0.017).

## Table S3. The Wilcoxon rank sum test of secondary outcomes (echocardiography) against preoperative assessment in CHD subtypes

In stratified analysis of different CHD subtypes, we observed significantly different echocardiographic profiles between the two groups. Specifically, for ASD patients, comparing with the preoperative assessment, the intervention group showed significant changes in left ventricular end diastolic dimension (LVDd), left ventricular end-systolic dimension (LVDs), and tricuspid blood flow velocity (TV) since the first postoperative follow-up, but had no changes in left ventricular posterior wall depth (LVPWd), left ventricular posterior wall thickness at end-systole (LVPWs), left ventricular ejection fraction (LVEF), main pulmonary artery diameter (MPA), and mitral blood flow velocity (MV) (Table S3a). The control group only had similar changes in LVDd and LVDs. For VSD patients, we observed significant decreases in LVDd, LVPWs, MPA, LVEF, and TV in the intervention group, but had no changes in LVDs, LVPWd and MV. In the control group, we only observed similar decreasing trends in MV (Table S3b). For PDA patients, the exercise program significantly decreased LVDd, LVDs, LVPWs, MPA, and MV in the intervention group. In the control group, only the 1 month assessment of LVDs and the 1, 3 month assessments of MPA showed significant decreases while others had no changes (Table S3c). For PS patients, the intervention group only showed significant changes in MPA and LVPWd, but the control group had significant decreases in LVDd, LVDs, LVPWDd, LVPWs, and MPA (Table S3d).

### Table S3a. The Wilcoxon rank sum test of secondary outcomes (echocardiography) for atrial septal defect (ASD) against preoperative assessment

|  | **Intervention group(n=19)** | | **Control group(n=19)** | |
| --- | --- | --- | --- | --- |
|  | **median (IQR)** | ***p* value** | **median (IQR)** | ***p* value** |
| **Left ventricular end diastolic dimension (LVDd)** | | | | |
| Baseline | 30.0 (27.8-33.0) | - | 30.9 (28.0-32.3) | - |
| 1 month | 33.6 (31.5-36.9) | .000* | 32.8 (30.3-34.1) | .003* |
| 3 months | 34.6 (30.1-36.1) | .000* | 33.1 (31.3-35.5) | .001* |
| 6 months | 34.9 (30.1-36.3) | .000* | 33.4 (31.5-36.0) | .000* |
| **Left ventricular end-systolic dimension (LVDs)** | | | | |
| Baseline | 18.7 (17.5-21.0) | - | 19.6 (18.6-20.7) | - |
| 1 month | 21.5 (19.3-22.4) | .004* | 20.1 (18.3-22.2) | .042* |
| 3 months | 22.2 (18.0-23.2) | .001* | 20.3 (18.8-22.2) | .035* |
| 6 months | 21.7 (19.3.-22.8) | .003* | 21.7 (19.3-22.3) | .002* |
| **Left ventricular posterior wall depth (LVPWDd)** | | | | |
| Baseline | 4.4 (4.1-5.0) | - | 4.1 (3.7-4.8) | - |
| 1 month | 4.8 (4.0-5.2) | .075 | 4.5 (3.8-4.8) | .405 |
| 3 months | 4.8 (3.9-5.6) | .317 | 4.6 (4.1-5.1) | .176 |
| 6 months | 5.3 (3.7-5.6) | .136 | 4.0 (3.7-4.6) | .380 |
| **Left ventricular posterior wall thickness at end-systole (LVPWs)** | | | | |
| Baseline | 7.3 (6.5-7.9) | - | 7.8 (6.3-8.6) | - |
| 1 month | 7.8 (7.4-8.2) | .076 | 6.9 (6.5-7.5) | .586 |
| 3 months | 7.3 (6.7-8.7) | .085 | 6.6 (6.3-7.7) | .492 |
| 6 months | 7.9 (6.7-9.1) | .305 | 7.1 (6.3-7.6) | .542 |
| **Left ventricular ejection fraction (LVEF)** | | | | |
| Baseline | 68.0 (64.0-70.0) | - | 67.0 (64.0-71.0) | - |
| 1 month | 67.0 (65.0-72.0) | .551 | 70.0 (67.0-72.0) | .551 |
| 3 months | 67.0 (63.0-70.0) | .789 | 70.0 (66.0-71.0) | .341 |
| 6 months | 68.0 (64.0-71.0) | .872 | 67.0 (64.0-70.0) | .630 |
| **Main pulmonary artery diameter (MPA)** | | | | |
| Baseline | 1.2 (1.2-1.5) | - | 1.2(1.0-1.9) | - |
| 1 month | 1.0 (0.9-1.2) | .013* | 1.2 (1.0-1.8) | .340 |
| 3 months | 1.0 (1.0-1.2) | .008* | 1.0 (1.0-1.2) | .029* |
| 6 months | 1.0 (1.0-1.2) | .071 | 1.0 (1.0-1.2) | .146 |
| **Mitral blood flow velocity (MV)** | | | | |
| Baseline | 1.0 (1.0-1.2) | - | 1.0 (1.0-1.2) | - |
| 1 month | 1.0 (1.0-1.2) | .974 | 1.0 (1.0-1.2) | .705 |
| 3 months | 1.0 (1.0-1.1) | .674 | 1.0 (1.0-1.2) | .457 |
| 6 months | 1.0 (1.0-1.0) | .914 | 1.0 (1.0-1.2) | .380 |
| **Tricuspid blood flow velocity (TV)** | | | | |
| Baseline | 0.9 (0.8-1.0) | - | 0.8 (0.8-1.0) | - |
| 1 month | 0.8 (0.8-0.8) | .020* | 0.8 (0.8-1.0) | .380 |
| 3 months | 0.8 (0.8-0.8) | .023* | 0.8 (0.8-0.8) | .142 |
| 6 months | 0.8 (0.8-0.8) | .020* | 0.8 (0.8-0.8) | .234 |
| **Z score of LVDd** | | | | |
| Baseline | -1.0 (-1.8- -0.4) | - | -0.7 (-0.9- -0.3) | - |
| 1 month | -0.1 (-0.7-0.4) | .001* | 0.0 (-0.6-0.2) | .044* |
| 3 months | -0.2 (-1.1-0.4) | .001* | -0.2 (-0.5-0.4) | .040* |
| 6 months | -0.3 (-0.8-0.6) | .004* | -0.1 (-0.6-0.5) | .013* |
| **Z score of LVDs** | | | | |
| Baseline | -0.5 (-1.1- -0.3) | - | -0.3 (-0.9-0.2) | - |
| 1 month | -0.1 (-0.7-0.8) | .008* | 0.1 (-0.3-0.3) | .117 |
| 3 months | 0.1 (-0.7-0.7) | .012* | -0.1 (-0.5-0.2) | .227 |
| 6 months | 0.0(-0.8-0.5) | .059 | 0.2 (-0.2-0.4) | .022* |
| **Z score of LVPWd** | | | | |
| Baseline | -0.1 (-0.6-0.3) | - | -0.3 (-0.5-0.5) | - |
| 1 month | 0.2 (-0.3-0.9) | .121 | 0.2 (-0.6-0.9) | .687 |
| 3 months | 0.1 (-0.7-0.9) | .809 | 0.3 (-0.4-0.8) | .615 |
| 6 months | 0.4 (-0.9-1.0) | .507 | -0.3 (-1.1-0.1) | .028* |
| **Z score of LVPWs** | | | | |
| Baseline | -1.3 (-1.9- -0.5) | - | -0.6 (-1.8-0.1) | - |
| 1 month | -0.6 (-0.8- -0.4) | .098 | -1.5 (-1.9- -0.2) | .559 |
| 3 months | -0.9 (-1.3- -0.4) | .459 | -1.7 (-2.4- -0.2) | .268 |
| 6 months | -0.9 (-1.5-0.2) | .528 | -1.3 (-2.4- -0.4) | .227 |
| **Z score of Ao** | | | | |
| Baseline | 3.2 (2.3-4.2) | - | 3.2 (2.7-3.6) | - |
| 1 month | 2.9 (2.1-3.6) | .227 | 3.4 (2.3-4.4) | .968 |
| 3 months | 3.4 (2.4-4.0) | .702 | 3.3 (2.9-4.0) | .872 |
| 6 months | 3.7 (3.0-4.2) | .658 | 3.2 (2.4-4.1) | .841 |

IQR: the interquartile range

* means significant difference(P<<0.017).

For ASD patients, comparing with the preoperative assessment, the intervention group showed significant changes in left ventricular end diastolic dimension (LVDd), left ventricular end-systolic dimension (LVDs), and tricuspid blood flow velocity (TV) since the first postoperative follow-up, but had no changes in left ventricular posterior wall depth (LVPWd), left ventricular posterior wall thickness at end-systole (LVPWs), left ventricular ejection fraction (LVEF), main pulmonary artery diameter (MPA), and mitral blood flow velocity (MV).The control group only had similar changes in LVDd and LVDs.

### Table S3b. The Wilcoxon rank sum test of secondary outcomes (echocardiography) for ventricular septal defect (VSD) against preoperative assessment

|  | **Intervention group(n=33)** | | **Control group(n=32)** | |
| --- | --- | --- | --- | --- |
|  | **median (IQR)** | ***p* value** | **median (IQR)** | ***p* value** |
| **Left ventricular end diastolic dimension (LVDd)** | | | | |
| Baseline | 36.1 (33.9-40.0) | - | 35.0 (33.4-38.2) | - |
| 1 month | 34.8 (32.4-36.5) | .000 | 34.4 (32.4-37.1) | .093 |
| 3 months | 35.0 (33.2-36.5) | .010 | 35.5 (32.7-36.8) | .044* |
| 6 months | 35.0 (32.4-36.4) | .004 | 35.1 (33.5-38.1) | .732 |
| **Left ventricular end-systolic dimension (LVDs)** | | | | |
| Baseline | 22.7 (21.4-24.8) | - | 23.0 (20.4-24.5) | - |
| 1 month | 22.4 (20.6-24.5) | .165 | 22.8 (21.5-24.5) | .500 |
| 3 months | 22.8 (20.4-24.5) | .332 | 22.7 (20.5-24.7) | .807 |
| 6 months | 23.0 (20.5-23.9) | .288 | 23.1 (21.2-24.6) | .366 |
| **Left ventricular posterior wall depth (LVPWDd)** | | | | |
| Baseline | 5.0 (4.4-5.6) | - | 4.8 (4.4-5.1) | - |
| 1 month | 4.6 (3.5-5.3) | .052 | 4.7 (3.9-5.5) | .984 |
| 3 months | 4.5 (3.8-5.2) | .058 | 4.7 (4.3-5.3) | .765 |
| 6 months | 4.6 (4.1-5.2) | .184 | 4.9 (3.8-5.7) | .520 |
| **Left ventricular posterior wall thickness at end-systole (LVPWs)** | | | | |
| Baseline | 8.8 (7.6-9.8) | - | 8.3 (7.3-8.8) | - |
| 1 month | 7.6 (6.6-9.0) | .001 | 7.4 (6.8-8.7) | .117 |
| 3 months | 7.5 (6.5-9.0) | .000 | 8.2 (7.3-9.1) | .931 |
| 6 months | 7.9 (6.6-9.0) | .003 | 8.1 (7.1-8.8) | .634 |
| **Left ventricular ejection fraction (LVEF)** | | | | |
| Baseline | 69.0 (66.0-71.0) | - | 66.5 (62.8-72.3) | - |
| 1 month | 65.0 (61.0-68.9) | .000 | 63.5 (61.0-66.8) | .009* |
| 3 months | 66.0 (62.5-71.0) | .002 | 64.5 (61.3-67.8) | .072 |
| 6 months | 65.0 (61.5-69.0) | .003 | 66.5 (64.0-68.8) | .222 |
| **Main pulmonary artery diameter (MPA)** | | | | |
| Baseline | 1.0 (1.0-1.2) | - | 1.0 (1.0-1.2) | - |
| 1 month | 1.0 (1.0-1.0) | .013 | 1.0 (0.9-1.2) | .099 |
| 3 months | 1.0 (1.0-1.2) | .046 | 1.0 (1.0-1.2) | .573 |
| 6 months | 1.0 (0.9-1.0) | .006 | 1.0 (1.0-1.2) | .355 |
| **Mitral blood flow velocity (MV)** | | | | |
| Baseline | 1.0 (1.0-1.2) | - | 1.2 (1.0-1.2) | - |
| 1 month | 1.0 (1.0-1.2) | .026 | 1.0 (1.0-1.2) | .018* |
| 3 months | 1.0 (1.0-1.0) | .090 | 1.0 (1.0-1.2) | .121 |
| 6 months | 1.0 (1.0-1.0) | .389 | 1.0 (1.0-1.2) | .002* |
| **Tricuspid blood flow velocity (TV)** | | | | |
| Baseline | 0.8 (0.8-0.8) | - | 0.8 (0.8-0.9) | - |
| 1 month | 0.8 (0.8-0.8) | .935 | 0.8 (0.8-0.8) | .863 |
| 3 months | 0.8 (0.8-0.8) | .874 | 0.8 (0.8-0.8) | .700 |
| 6 months | 0.8 (0.8-1.0) | .034 | 0.8 (0.8-0.8) | .655 |
| **Z score of LVDd** | | | | |
| Baseline | 0.5 (0.0-1.6) | - | 0.6 (0.0-1.1) | - |
| 1 month | 0.1 (-0.5-0.4) | .000 | 0.4 (-0.6-0.8) | .008 |
| 3 months | 0.0 (-0.4-0.7) | .002 | 0.1 (-0.2-0.5) | .012* |
| 6 months | -0.1 (-0.6-0.3) | .000 | 0.1 (-0.3-0.7) | .041* |
| **Z score of LVDs** | | | | |
| Baseline | 0.6 (0.0-1.4) | - | 0.6 (-0.2-1.2) | - |
| 1 month | 0.2 (-0.2-1.2) | .169 | 0.7 (0.1-1.1) | .911 |
| 3 months | 0.4 (0.0-1.0) | .231 | 0.4 (-0.1-1.0) | .531 |
| 6 months | 0.3 (-0.1-0.7) | .028 | 0.5 (-0.1-0.7) | .531 |
| **Z score of LVPWd** | | | | |
| Baseline | 0.5 (0.1-1.0) | - | 0.3 (-0.2-0.7) | - |
| 1 month | 0.1 (-1.2-0.9) | .046 | 0.2 (-0.7-1.0) | .874 |
| 3 months | 0.0 (-0.7-0.6) | .043 | 0.3 (-0.3-0.7) | .695 |
| 6 months | 0.1 (-0.7-0.4) | .029 | 0.4 (-0.6-0.8) | .993 |
| **Z score of LVPWs** | | | | |
| Baseline | 0.2 (-0.6-0.7) | - | -0.6 (-1.1-0.4) | - |
| 1 month | -1.0 (-1.9- -0.1) | .001 | -0.9 (-1.7- -0.4) | .046* |
| 3 months | -0.9 (-1.6- -0.2) | .000 | -0.4 (-1.4-0.2) | .593 |
| 6 months | -0.9 (-1.9- -0.2) | .000 | -0.5 (-1.5- -0.1) | .210 |
| **Z score of Ao** | | | | |
| Baseline | 3.5 (2.5-4.0) | - | 3.1 (2.4-4.0) | - |
| 1 month | 3.7 (2.5-4.2) | .866 | 3.5 (2.9-4.2) | .295 |
| 3 months | 3.5 (2.8-4.7) | .077 | 2.6 (3.3-4.1) | .701 |
| 6 months | 3.2 (2.5-4.2) | .851 | 3.4 (2.7-4.0) | .454 |

IQR: the interquartile range

* means significant difference(P<0.017).

For VSD patients, we observed significant decreases in left ventricular end diastolic dimension (LVDd), left ventricular posterior wall thickness at end-systole (LVPWs) main pulmonary artery diameter (MPA), left ventricular ejection fraction (LVEF), and tricuspid blood flow velocity (TV) in the intervention group, but had no changes in left ventricular end-systolic dimension (LVDs), left ventricular posterior wall depth (LVPWd) and mitral blood flow velocity (MV). In the control group, we only observed similar decreasing trends in MV.

### Table S3c. The Wilcoxon rank sum test of secondary outcomes (echocardiography) for patent ductus arteriosis (PDA) against preoperative assessment

|  | **Intervention group(n=31)** | | **Control group(n=32)** | |
| --- | --- | --- | --- | --- |
|  | **median (IQR)** | ***p* value** | **median (IQR)** | ***p* value** |
| **Left ventricular end diastolic dimension (LVDd)** | | | | |
| Baseline | 33.5 (31.6-36.3) | - | 32.0 (30.5-40.6) | - |
| 1 month | 31.8 (29.0-33.8) | .021* | 31.4 (29.6-34.7) | .018* |
| 3 months | 32.0 (29.0-33.4) | .008* | 31.8 (29.4-34.3) | .078 |
| 6 months | 31.8 (29.6-33.6) | .012* | 31.4 (29.1-34.1) | .111 |
| **Left ventricular end-systolic dimension (LVDs)** | | | | |
| Baseline | 21.2 (20.1-23.9) | - | 21.8 (18.9-26.3) | - |
| 1 month | 20.3 (19.0-22.4) | .022* | 20.4 (18.3-23.7) | .016* |
| 3 months | 20.3 (18.1-22.4) | .006* | 20.3 (18.2-28.3) | .178 |
| 6 months | 20.1 (18.7-21.9) | .032* | 20.4 (17.8-22.9) | .058 |
| **Left ventricular posterior wall depth (LVPWDd)** | | | | |
| Baseline | 4.5 (4.0-5.1) | - | 4.4 (3.9-5.1) | - |
| 1 month | 4.6 (4.1-5.6) | .590 | 4.6 (3.6-5.1) | .158 |
| 3 months | 4.3 (3.9-4.8) | .204 | 4.2 (3.6-5.1) | .141 |
| 6 months | 4.1 (3.6-4.8) | .083 | 4.6 (3.8-5.4) | .761 |
| **Left ventricular posterior wall thickness at end-systole (LVPWs)** | | | | |
| Baseline | 7.6 (7.0-8.7) | - | 7.1 (6.5-8.0) | - |
| 1 month | 7.6 (6.7-8.7) | .786 | 7.0 (6.0-8.1) | .671 |
| 3 months | 7.2 (6.5-7.6) | .113 | 7.2 (5.8-7.9) | .437 |
| 6 months | 6.9 (6.4-7.8) | .004* | 7.0 (6.5-8.3) | .981 |
| **Left ventricular ejection fraction (LVEF)** | | | | |
| Baseline | 67.0 (63.0-71.0) | - | 65.5 (63.0-67.0) | - |
| 1 month | 65.0 (61.0-70.0) | .602 | 64.0 (61.3-71.8) | .898 |
| 3 months | 67.0 (63.0-70.0) | .588 | 64.5 (61.3-71.8) | .933 |
| 6 months | 67.0 (61.0-71.0) | .797 | 67.0 (63.3-71.5) | .434 |
| **Main pulmonary artery diameter (MPA)** | | | | |
| Baseline | 1.0 (1.0-1.2) | - | 1.2 (1.0-1.2) | - |
| 1 month | 1.0 (1.0-1.2) | .295 | 1.0 (0.9-1.2) | .006* |
| 3 months | 1.0 (1.0-1.2) | .378 | 1.0 (1.0-1.2) | .037* |
| 6 months | 1.0 (1.0-1.0) | .002* | 1.0 (1.0-1.2) | .176 |
| **Mitral blood flow velocity (MV)** | | | | |
| Baseline | 1.0 (1.0-1.2) | - | 1.0 (1.0-1.1) | - |
| 1 month | 1.0 (1.0-1.2) | .310 | 1.0 (1.0-1.1) | .623 |
| 3 months | 1.0 (1.0-1.0) | .055 | 1.0 (1.0-1.0) | .731 |
| 6 months | 1.0 (1.0-1.0) | .042* | 1.0 (1.0-1.2) | .417 |
| **Tricuspid blood flow velocity (TV)** | | | | |
| Baseline | 0.8 (0.8-1.0) | - | 0.8 (0.8-0.8) | - |
| 1 month | 0.8 (0.8-0.8) | .528 | 0.8 (0.8-0.8) | .593 |
| 3 months | 0.8 (0.8-0.8) | .210 | 0.8 (0.8-0.8) | .705 |
| 6 months | 0.8 (0.8-0.8) | .060 | 0.8 (0.8-1.0) | .248 |
| **Z score of LVDd** | | | | |
| Baseline | 0.9 (0.0-2.9) | - | 1.5 (-0.3-2.5) | - |
| 1 month | 0.2 (-0.4-1.9) | .001* | 0.7 (0.0-1.1) | .004* |
| 3 months | 0.4 (-0.3-1.5) | .000* | 0.4 (0.1-1.0) | .003* |
| 6 months | 0.2 (-0.3-0.7) | .000* | 0.4 (-0.3-0.9) | .000* |
| **Z score of LVDs** | | | | |
| Baseline | 0.9 (0.4-2.6) | - | 1.7 (0.2-2.6) | - |
| 1 month | 0.7 (-0.2-2.4) | .003* | 1.1 (0.2-1.7) | .004* |
| 3 months | 0.5 (-0.1-1.4) | .001* | 0.7 (0.3-1.2) | .007* |
| 6 months | 0.5 (-0.5-1.1) | .001* | 0.4 (-0.1-1.5) | .001* |
| **Z score of LVPWd** | | | | |
| Baseline | 0.6 (-0.2-1.7) | - | 0.4 (0.0-1.2) | - |
| 1 month | 0.7 (-0.2-1.5) | .658 | 0.2 (-0.4-1.3) | .130 |
| 3 months | 0.4 (-0.3-0.8) | .159 | -0.1 (-0.8-1.2) | .017* |
| 6 months | 0.1 (-0.9-0.6) | .010* | 0.4 (-0.2-1.1) | .114 |
| **Z score of LVPWs** | | | | |
| Baseline | -0.2 (-0.7-0.9) | - | -0.5 (-1.7-0.2) | - |
| 1 month | -0.4 (-1.5-0.6) | .254 | -0.5 (-1.5-0.2) | .701 |
| 3 months | -0.7 (-1.3-0.0) | .009* | -0.7 (-1.7-0.1) | .119 |
| 6 months | -0.7 (-1.4- -0.3) | .000* | -1.3 (-0.7-0.1) | .197 |
| **Z score of Ao** | | | | |
| Baseline | 3.7 (2.7-5.5) | - | 4.1 (3.3-5.0) | - |
| 1 month | 3.9 (2.4-4.8) | .267 | 4.2 (3.3-5.2) | .519 |
| 3 months | 3.8 (2.8-5.0) | .943 | 3.9 (2.7-5.2) | .938 |
| 6 months | 4.2 (2.9-5.0) | .551 | 3.9 (3.4-4.9) | .837 |

IQR: the interquartile range

* means significant difference(P<0.017).

### Table S3d. The Wilcoxon rank sum test of secondary outcomes (echocardiography)y for pulmonary stenosis (PS) against preoperative assessment

|  | **Intervention group(n=12)** | | **Control group(n=14)** | |
| --- | --- | --- | --- | --- |
|  | **median (IQR)** | ***p* value** | **median (IQR)** | ***p* value** |
| **Left ventricular end diastolic dimension (LVDd)** | | | | |
| Baseline | 24.9 (22.5-29.4) | - | 24.8 (21.5-32.2) | - |
| 1 month | 24.2 (23.2-29.6) | .066 | 26.2 (24.4-31.1) | .402 |
| 3 months | 26.9 (24.8-31.8) | .004* | 28.4 (24.8-31.8) | .005* |
| 6 months | 26.9 (24.7-30.3) | .150 | 28.6 (26.3-32.2) | .002* |
| **Left ventricular end-systolic dimension (LVDs)** | | | | |
| Baseline | 16.2 (14.4-18.9) | - | 16.2 (13.5-19.4) | - |
| 1 month | 15.6 (14.5-18.8) | .807 | 17.0 (14.7-18.8) | .638 |
| 3 months | 15.9 (15.1-20.1) | .248 | 18.2 (15.5-20.6) | .002* |
| 6 months | 16.9 (15.0-20.7) | .532 | 18.7 (14.8-19.3) | .003* |
| **Left ventricular posterior wall depth (LVPWDd)** | | | | |
| Baseline | 4.0 (2.4-4.9) | - | 3.1 (2.6-3.9) | - |
| 1 month | 4.2 (3.5-5.1) | .118 | 3.8 (3.1-4.7) | .031* |
| 3 months | 3.6 (3.1-4.6) | .585 | 3.8 (3.6-4.9) | .010* |
| 6 months | 4.6 (3.5-5.1) | .025* | 4.0 (3.8-4.5) | .038* |
| **Left ventricular posterior wall thickness at end-systole (LVPWs)** | | | | |
| Baseline | 6.3 (5.1-8.1) | - | 5.5 (4.6-6.9) | - |
| 1 month | 6.9 (5.5-8.2) | .956 | 6.7 (5.3-7.5) | .068 |
| 3 months | 6.3 (5.6-7.5) | .823 | 6.2 (5.9-7.4) | .002* |
| 6 months | 6.7 (6.4-7.5) | .532 | 6.3 (5.8-7.9) | .044* |
| **Left ventricular ejection fraction (LVEF)** | | | | |
| Baseline | 70.0 (68.0-72.8) | - | 69.5 (65.8-70.3) | - |
| 1 month | 68.5 (63.3-71.0) | .397 | 68.5 (64.5-71.5) | .877 |
| 3 months | 71.0 (65.0-74.5) | .918 | 66.0 (61.8-73.3) | .487 |
| 6 months | 69.0 (64.8-75.8) | .747 | 67.5 (61.0-73.8) | .746 |
| **Main pulmonary artery diameter (MPA)** | | | | |
| Baseline | 3.5 (2.7-4.1) | - | 3.9 (2.9-4.7) | - |
| 1 month | 3.5 (2.7-4.1) | .025* | 2.5 (1.8-3.1) | .013* |
| 3 months | 2.7 (2.3-3.4) | .004* | 2.3 (1.9-2.9) | .013* |
| 6 months | 2.7 (2.1-3.1) | .004* | 3.1 (1.7-2.4) | .005* |
| **Mitral blood flow velocity (MV)** | | | | |
| Baseline | 1.0 (1.0-1.2) | - | 1.0 (1.0-1.1) | - |
| 1 month | 1.0 (0.9-1.1) | .518 | 1.1 (1.0-1.2) | .083 |
| 3 months | 1.0 (0.9-1.2) | .785 | 1.0 (1.0-1.0) | .705 |
| 6 months | 1.0 (1.0-1.0) | .666 | 1.0 (1.0-1.0) | .564 |
| **Tricuspid blood flow velocity (TV)** | | | | |
| Baseline | 0.8 (0.8-1.0) | - | 0.8 (0.8-1.0) | - |
| 1 month | 0.8 (0.8-1.0) | .705 | 0.8 (0.8-1.0) | .527 |
| 3 months | 0.8 (0.8-0.8) | .131 | 0.8 (0.8-1.0) | .888 |
| 6 months | 0.8 (0.8-1.0) | .705 | 0.8 (0.8-1.0) | .705 |
| **Z score of LVDd** | | | | |
| Baseline | -0.9 (-0.5-0.2) | - | 0.0 (-0.6-1.0) | - |
| 1 month | -0.4 (-0.8-0.2) | .844 | -0.1 (-0.8-1.0) | .778 |
| 3 months | 0.0 (-0.7-0.3) | .530 | 0.2 (-0.4-0.8) | .875 |
| 6 months | -0.7 (-1.4- -0.1) | .388 | 0.1 (-0.4-0.5) | .826 |
| **Z score of LVDs** | | | | |
| Baseline | 0.0 (-0.8-1.1) | - | 0.1 (-0.5-1.0) | - |
| 1 month | 0.1 (-1.1-0.7) | .754 | 0.1 (-1.1-1.6) | .826 |
| 3 months | 0.0 (-0.9-0.4) | .814 | 0.5 (-0.1-1.1) | .331 |
| 6 months | -0.5 (-1.5-0.3) | .209 | 0.3 (-0.5-0.9) | .851 |
| **Z score of LVPWd** | | | | |
| Baseline | 0.2 (-1.7-1.0) | - | -0.6 (-1.2-0.1) | - |
| 1 month | 0.5 (-0.1-0.9) | .308 | -0.1 (-0.6-0.8) | .028* |
| 3 months | -0.4 (-0.9-0.9) | 1.000 | 0.2 (-0.2-0.6) | .026* |
| 6 months | 0.2 (-0.5-1.2) | .084 | 0.2 (-0.1-0.5) | .221 |
| **Z score of LVPWs** | | | | |
| Baseline | -0.8 (-1.6- -0.1) | - | -1.2 (-2.3- -0.6) | - |
| 1 month | -0.8 (-1.3-0.2) | .583 | 0.0 (-1.4-0.4) | .109 |
| 3 months | -0.9 (-2.2-0.0) | .875 | -0.9 (-1.2-0.1) | .019* |
| 6 months | -0.7 (-1.1-0.2) | .638 | -1.3 (-2.0-0.3) | .778 |
| **Z score of Ao** | | | | |
| Baseline | 3.4 (2.7-3.9) | - | 3.0 (2.2-4.4) | - |
| 1 month | 2.8 (1.7-3.3) | .209 | 3.1 (2.0-3.9) | .778 |
| 3 months | 3.1 (2.4-3.7) | .209 | 2.5 (3.8-4.9) | .158 |
| 6 months | 3.1 (2.6-3.6) | .583 | 3.6 (3.0-4.5) | .140 |

IQR: the interquartile range

* means significant difference(P<0.017).

## Table S4. The Wilcoxon rank sum test of secondary outcomes (modified Ross score) against preoperative assessment

In this table, we compared postoperative assessments against the preoperative assessment separately in the intervention group and in the control group.

|  | **Intervention group (n=95)** | | **Control group (n=97)** | |
| --- | --- | --- | --- | --- |
|  | **median (IQR)** | ***p* value** | **median (IQR)** | ***p* value** |
| Baseline | 1.0 (0.0-1.0) | - | 1.0 (0.0-1.0) | - |
| 1 month | 1.0 (0.0-1.0) | .791 | 1.0 (0.0-1.0) | .123 |
| 3 months | 1.0 (0.0-1.0) | .975 | 1.0 (0.0-1.0) | .456 |
| 6 months | 1.0 (0.0-1.0) | .717 | 1.0 (0.0-1.0) | .938 |

IQR: the interquartile range

Table S5. The Wilcoxon rank sum test of secondary outcomes (parents’ anxiety, burden and quality of life) against preoperative assessment

|  | **Intervention group**  **(n=93)** | | **Control group**  **(n=95)** | |
| --- | --- | --- | --- | --- |
|  | **median (IQR)** | ***p* value** | **median (IQR)** | ***p* value** |
| **SAS of rude score** | | | | |
| Baseline | 30.0 (26.5-37.0) | - | 33.0 (27.0-39.0) | - |
| 1 month | 32.0 (27.0-36.0) | 0.590 | 33.0 (27.0-36.0) | 0.477 |
| 3 months | 32.0 (25.5-36.0) | 0.607 | 32.0 (26.0-37.0) | 0.119 |
| 6 months | 33.0 (28.0-38.0) | 0.028 | 34.0 (29.0-38.0) | 0.201 |
| **SAS of standard score** | | | | |
| Baseline | 37.5 (33.1-46.3) | - | 41.3 (33.8-48.8) | - |
| 1 month | 40.0 (33.8-45.0) | 0.590 | 41.3 (33.8-45.0) | 0.477 |
| 3 months | 40.0 (31.8-45.0) | 0.607 | 40.0 (32.5-46.3) | 0.119 |
| 6 months | 41.3 (35.0-47.5) | 0.028 | 42.5 (36.3-47.5) | 0.201 |
| **ZCBS score** |  |  |  |  |
| Baseline | 22.0 (14.0-30.0) | - | 22.5 (14.3-30.0) | - |
| 1 month | 23.0 (13.0-33.3) | 0.367 | 23.5 (16.0-34.0) | 0.188 |
| 3 months | 23.0 (15.0-33.0) | 0.227 | 22.0 (13.0-32.0) | 0.632 |
| 6 months | 18.5 (11.0-31.0) | 0.103 | 23.5 (17.0-34.0) | 0.206 |
| **SF-36 of total score** | | | | |
| Baseline | 640.0 (552.5-706.5) | - | 629.0 (532.5-686.0) | - |
| 1 month | 641.0 (536.0-707.5) | 0.843 | 623.0 (502.5-677.0) | 0.538 |
| 3 months | 666.0 (561.5-708.5) | 0.364 | 627.0 (515.5-688.5) | 0.827 |
| 6 months | 678.0 (611.5-733.5) | 0.001 | 629.0 (519.5-683.5) | 0.888 |
| **SF-36 of physical component score** | | | | |
| Baseline | 339.0 (304.0-369.0) | - | 348.0 (288.0-370.0) | - |
| 1 month | 344.0 (293.0-374.0) | 0.948 | 329.0 (277.5-362.0) | 0.185 |
| 3 months | 347.0 (306.0-379.0) | 0.498 | 329.0 (282.0-361.5) | 0.257 |
| 6 months | 359.0 (328.0-383.0) | 0.002 | 338.0 (292.5-360.0) | 0.896 |
| **SF-36 of mental component score** | | | | |
| Baseline | 308.0 (242.5-308.0) | - | 277.0 (237.5-327.0) | - |
| 1 month | 302.0 (241.5-335.0) | 0.720 | 291.0 (233.0-328.0) | 0.860 |
| 3 months | 310.0 (255.5-337.0) | 0.303 | 294.0 (243.0-328.5) | 0.629 |
| 6 months | 326.0 (287.5-349.5) | 0.002 | 291.0 (224.5-325.0) | 0.890 |

Abbreviations: IQR, the interquartile range; SAS, Self-Rating Anxiety Scale; SF-36, 36-item short-form health survey; ZCBS, Zarit Caregiver Burden Scale.

## Table S6. Characteristics of the enrolled CHD children with various residencies at baseline (n=192)

|  | **Shanghai group** | | **Non-Shanghai group** | |
| --- | --- | --- | --- | --- |
|  | **No. of Patients** | **Value** | **No. of Patients** | **Value** |
| **Age, month** | 57 | 35.0 (11.0-47.0) | 135 | 32.0 (18.0-44.0) |
| **Sex** | | | | |
| Male | 24 | 24 (42.1%) | 61 | 61 (45.2%) |
| Female | 33 | 33 (57.9%) | 74 | 74 (54.8%) |
| **Type of CHD** | | | | |
| PDA | 21 | 21 (36.8%) | 42 | 42 (31.1%) |
| PS | 9 | 9 (15.8%) | 17 | 17 (12.6%) |
| VSD | 16 | 16 (28.1%) | 49 | 49 (36.3%) |
| ASD | 11 | 11 (19.3%) | 27 | 27 (20.0%) |
| **Motor developmental quotient** | | | | |
| GMQ | 57 | 91.0 (85.0-96.0) | 135 | 91.0 (85.0-96.0) |
| FMQ | 57 | 94.0 (91.0-100.0) | 135 | 94.0 (91.0-100.0) |
| TMQ | 57 | 93.0 (88.0-97.0) | 135 | 92.0 (89.0-96.0) |
| **Modified Ross score** | | | | |
| 0 | 24 | 24 (42.1%) | 52 | 52 (38.5%) |
| 1 | 24 | 24 (42.1%) | 65 | 65 (48.1%) |
| 2 | 9 | 9 (15.8%) | 18 | 18 (13.3%) |
| **Cardiac structural indexes** | | | | |
| LVDd, mm | 57 | 32.6 (29.1-37.1) | 135 | 33.0 (29.4-36.0) |
| LVDs, mm | 57 | 21.2 (19.2-23.9) | 135 | 21.0 (18.6-23.1) |
| LVPWd, mm | 57 | 4.6 (4.0-5.1) | 135 | 4.5 (3.8-5.1) |
| LVPWs, mm | 57 | 7.7 (7.0-9.0) | 132 | 7.5 (6.5-8.7) |
| LVEF, % | 57 | 69.0 (65.0-70.5) | 135 | 67.0 (64.0-71.0) |
| MPA, % | 57 | 1.2 (1.0-1.4) | 135 | 1.2 (1.0-1.4) |
| MV, m/s | 57 | 1.0 (1.0-1.2) | 135 | 1.0 (1.0-1.2) |
| TV, m/s | 57 | 0.8 (0.8-1.0) | 135 | 0.8 (0.8-0.9) |
| **Bone quality** | | | | |
| SOS, m/s | 57 | 3367.0 (3103.5-3550.5) | 133 | 3406.0 (3264.5-3524.0) |
| Bone strength percentile | 57 | 46.0 (29.5-73.5) | 133 | 53.0 (22.5-81.5) |
| Z score | 57 | -0.1 (-0.6-0.6) | 133 | 0.1 (-0.8-0.9) |
| **SAS score** | | | | |
| Rude score | 55 | 30.0 (25.0-36.0) | 133 | 32.0 (27.5-38.0) |
| Standard score | 55 | 37.5 (31.3-45.0) | 133 | 40.0 (34.4-47.5) |
| **ZCBS score** | 57 | 23.0 (15.5-31.0) | 133 | 22.0 (14.0-30.0) |
| **SF-36 score** | | | | |
| PCS | 57 | 340.0 (278.5-368.0) | 135 | 340.0 (304.0-370.0) |
| MCS | 57 | 290.0 (246.5-329.5) | 135 | 284.0 (227.0-332.0) |
| Total score | 57 | 629.0 (526.5-695.0) | 135 | 628.0 (531.0-705.0) |
| **Physical development indicators** | | | | |
| Weight, kg | 57 | 15.0 (9.2-17.0) | 135 | 14.0 (10.0-16.5) |
| Height, cm | 57 | 95.0 (72.3-105.0) | 135 | 92.0 (80.0-100.7) |
| BMI, kg/m^2^ | 57 | 16.5 (15.8-17.3) | 135 | 16.1 (15.0-17.6) |
| Upper-arm circumference, cm | 57 | 16.0 (15.0-17.0) | 134 | 16.0 (15.0-17.0) |
| Head circumference, cm | 57 | 49.0 (45.0-50.0) | 134 | 48.0 (45.4-49.8) |
| Chest circumference, cm | 57 | 50.0 (46.3-53.0) | 134 | 50.0 (47.0-54.0) |

Data are n (%), median (IQR). Ao=Aort. CHD= Congenital heart disease. PDA= Patent ductus arteriosis. PS= Pulmonary stenosis. VSD= Ventricular septal defect. ASD= Atrial septal defect. GMQ= gross motor quotient. FMQ= fine motor quotient. TMQ= total motor quotient. LVDd=left ventricular end diastolic dimension. LVDs= left ventricular end-systolic dimension. LVPWd=left ventricular posterior wall depth. LVPWs= left ventricular posterior wall thickness at end-systole. LVEF= left ventricular ejection fraction. MPA= main pulmonary artery diameter. MV= mitral blood flow velocity. TV= tricuspid blood flow velocity. SOS= speed of sound. SAS= Self-Rating Anxiety Scale. ZCBS= Zarit Caregiver Burden Scale. SF-36= Short Form 36-item Health Survey. PCS= physical component summary. MCS= mental component summary. BMI= body mass index.

## Table S7. Outcomes of the CHD children with various residencies in the intervention group and the control group separately

Approximately 70% of patients in this study were from other regions outside Shanghai, we tested if there were significant differences in the preoperative assessment findings among the CHD patients between the Shanghai residents and non-Shanghai residents in the intervention group or the control group separately. In this study, participating families who did not live locally only traveled to Shanghai for catheterization and postoperative assessments; daily rehabilitating exercises during the 6-month study period were completed under the remote guidance and supervision of the study team in Shanghai via WeChat and phone calls.

### Table S7a. Outcomes of the CHD children with various residencies in the control group at 6 months after catheterization (n=97)

|  | | **Shanghai group** | | **Non-Shanghai group** | |
| --- | --- | --- | --- | --- | --- |
|  |  | **No. of Patients** | **Value** | **No. of Patients** | **Value** |
| **Motor developmental quotient** | | | | | |
| GMQ | | 27 | 89.0 (83.0-98.0) | 70 | 91.0 (87.0-96.5) |
| FMQ | | 27 | 91.0 (87.0-96.5) | 70 | 100.0 (94.0-106.0) |
| TMQ | | 27 | 94.0 (89.0-96.0) | 70 | 94.0 (92.0-98.0) |
| **Modified Ross score** | | | | | |
| 0 | | 14 | 14 (51.9%) | 26 | 26 (37.1%) |
| 1 | | 9 | 9 (33.3%) | 32 | 32 (45.7%) |
| 2 | | 4 | 4 (14.8%) | 12 | 12 (17.2%) |
| **Bone quality** | | | | | |
| SOS, m/s | | 27 | 3438.0 (3228.0-3543.0) | 70 | 3421.0 (3299.5-3547.5) |
| Bone strength percentile | | 27 | 46.0 (33.0-79.0) | 70 | 49.5 (19.3-85.3) |
| Z score | | 27 | -0.1 (-0.5-0.8) | 70 | 0.0 (-0.9-1.0) |
| **SAS score** | | | | | |
| Rude score | | 26 | 33.5 (28.0-37.3) | 69 | 34.0 (30.0-38.0) |
| Standard score | | 26 | 41.9 (35.0-46.6) | 69 | 42.5 (37.5-47.5) |
| **ZCBS score** | 28 | 23.0 (14.0-34.0) | 68 | 24.0 (17.0-34.8) |  |
| **SF-36 score** | | | | |  |
| PCS | | 27 | 80.8 (66.4-89.0) | 66 | 85.3 (77.9-90.2) |
| MCS | | 27 | 72.4 (59.7-81.0) | 66 | 74.3 (56.3-81.8) |
| Total score | | 27 | 75.5 (65.0-83.2) | 66 | 79.3 (66.1-86.0) |
| **Physical development indicators** | | | | | |
| Weight, kg | | 27 | 13.8 (11.1-18.5) | 70 | 15.0 (11.3-17.6) |
| Height, cm | | 27 | 98.0 (80.5-109.0) | 70 | 98.5 (85.0-108.2) |
| BMI, kg/m^2^ | | 27 | 15.9 (14.7-17.4) | 70 | 15.5 (14.7-17.0) |
| Upper-arm circumference, cm | | 27 | 17.0 (15.0-18.0) | 70 | 16.0 (15.0-17.3) |
| Head circumference, cm | | 27 | 48.0 (45.0-50.0) | 70 | 49.0 (47.0-50.0) |
| Chest circumference, cm | | 27 | 52.0 (48.0-54.0) | 70 | 52.0 (49.0-55.0) |

Data are n (%), median (IQR). Ao=Aort. CHD= Congenital heart disease. PDA= Patent ductus rteriosis. PS= Pulmonary stenosis. VSD= Ventricular septal defect. ASD= Atrial septal defect. GMQ= gross motor quotient. FMQ= fine motor quotient. TMQ= total motor quotient. LVDd=left ventricular end diastolic dimension. LVDs= left ventricular end-systolic dimension. LVPWd=left ventricular posterior wall depth. LVPWs= left ventricular posterior wall thickness at end-systole. LVEF= left ventricular ejection fraction. LVFS= left ventricular fractional shortening. MPA= main pulmonary artery diameter. MV= mitral blood flow velocity. TV= tricuspid blood flow velocity. SOS= speed of sound. SAS= Self-Rating Anxiety Scale. ZCBS= Zarit Caregiver Burden Scale. SF-36= Short Form 36-item Health Survey. PCS= physical component summary. MCS= mental component summary. BMI= body mass index.

### Table S7b. Outcomes of the CHD children with various residencies in the intervention group at 6 months after catheterization (n=95)

|  | **Shanghai group** | | **Non-Shanghai group** | |
| --- | --- | --- | --- | --- |
|  | **No. of Patients** | **Value** | **No. of Patients** | **Value** |
| **Motor developmental quotient** | | | | |
| GMQ | 30 | 98.0 (92.5-100.0) | 65 | 96.0 (91.0-100.0) |
| FMQ | 30 | 106.0 (100.0-109.0) | 65 | 103.0 (97.0-109.0) |
| TMQ | 30 | 100.0 (94.0-104.0) | 65 | 97.0 (94.0-103.0) |
| **Modified Ross score** | | | | |
| 0 | 13 | 13 (43.4%) | 24 | 24 (36.9%) |
| 1 | 10 | 10 (33.3%) | 32 | 32 (49.2%) |
| 2 | 7 | 7 (23.3%) | 9 | 9 (13.9%) |
| **Bone quality** | | | | |
| SOS, m/s | 30 | 3468.0 (3203.8-3570.5) | 63 | 3388.0 (3278.0-3526.0) |
| Bone **s**trength percentile | 30 | 56.5 (32.0-77.0) | 63 | 50.0 (25.0-78.0) |
| Z score | 30 | 0.2 (-0.5-0.7) | 63 | 0.0 (-0.7-0.8) |
| **SAS score** | | | | |
| Rude score | 28 | 33.5 (28.0-37.8) | 65 | 33.0 (28.5-38.0) |
| Standard score | 28 | 41.9 (35.0-47.2) | 65 | 41.3 (35.6-47.5) |
| **ZCBS score** | 30 | 20.5 (9.5-35.3) | 64 | 18.0 (13.0-27.8) |
| **SF-36 score** | | | | |
| PCS | 29 | 91.3 (81.1-95.9) | 64 | 89.8 (82.6-95.6) |
| MCS | 29 | 81.5 (66.0-88.8) | 64 | 82.1 (73.0-87.1) |
| Total score | 29 | 84.9 (73.9-91.8) | 64 | 84.7 (76.6-91.7) |
| **Physical development indicators** | | | | |
| Weight, kg | 30 | 15.1 (11.7-19.9) | 65 | 14.7 (11.4-19.0) |
| Height, cm | 30 | 101.0 (79.8-110.2) | 65 | 98.2 (84.0-108.3) |
| BMI, kg/m^2^ | 30 | 16.4 (15.5-18.2) | 65 | 16.3 (15.5-17.1) |
| Upper-arm circumference, cm | 30 | 17.0 (15.9-18.1) | 65 | 17.0 (15.0-18.0) |
| Head circumference, cm | 30 | 49.8 (47.9-51.0) | 65 | 48.5 (46.5-50.0) |
| Chest circumference, cm | 30 | 51.0 (48.9-55.0) | 65 | 52.0 (49.0-55.0) |

Data are n (%), median (IQR). Ao=Aort. CHD= Congenital heart disease. PDA= Patent ductus arteriosis. PS= Pulmonary stenosis. VSD= Ventricular septal defect. ASD= Atrial septal defect. GMQ= gross motor quotient. FMQ= fine motor quotient. TMQ= total motor quotient. LVDd=left **ventricular** end diastolic dimension. LVDs= left ventricular end-systolic dimension. LVPWd=left ventricular posterior wall depth. LVPWs= left ventricular posterior wall thickness at end-systole. LVEF= left ventricular ejection fraction. LVFS= left ventricular fractional shortening. MPA= main pulmonary artery diameter. MV= mitral blood flow velocity. TV= tricuspid blood flow velocity. SOS= speed of sound. SAS= Self-Rating Anxiety Scale. ZCBS= Zarit Caregiver Burden Scale. SF-36= Short Form 36-item Health Survey. PCS= physical component summary. MCS= mental component summary. BMI= body mass index.
